# Study of the Gas-Phase Pyrolysis of N-Aryl-3-Oxobutanamides and the 2-Aryl Hydrazone Derivatives: A Novel Computational Approach Including DFT with Multivariant Analysis

**DOI:** 10.3390/molecules31183201

**Published:** 2026-09-10

**Authors:** Rosalinda Ipanaque-Chávez, Marcos Loroño, Tania Cordova-Sintjago, José L. Paz, Cecilio Julio Alberto Garrido Schaeffer

**Affiliations:** 1 Departamento Académico de Fisicoquímica, Facultad de Química e Ingeniería Química, Universidad Nacional Mayor de San Marcos, Lima 15081, Peru; rosalinda.ipanaque@unmsm.edu.pe; 2 Department of Natural Sciences, Santa Fe College, Gainesville, FL 32066, USA; tania.cordova-sintjago@sfcollege.edu; 3 Departamento Académico de Química Inorgánica, Facultad de Química e Ingeniería Química, Universidad Nacional Mayor de San Marcos, Lima 15081, Peru; jpazr@unmsm.edu.pe; 4 Departamento Académico de Operaciones Unitarias, Facultad de Química e Ingeniería Química, Universidad Nacional Mayor de San Marcos, Lima 15081, Peru; cgarridos@unmsm.edu.pe

**Keywords:** DFT calculations, N-aryl-3-oxobutanamides (β-ketoamides), N-arylhydrazone, reaction mechanism, principal component analysis (PCA), dynamic synchronicity *SyD*

## Abstract

In this work, we carried out a computational study of the gas-phase thermal decomposition of N-aryl-**3**-oxobutanamides (β-ketoamides) and the 2-arylhydrazone derivatives. We performed calculations using density functional theory (DFT) at the B97D-GD3BJ/deft2tzvp level, with multivariant analysis including descriptors of global reactivity, e.g., ionization energy (I), electron affinity (A), molecular hardness (η) and electrophilicity (ω). The objective was to elucidate the reaction mechanism. To this end, we modeled the structures of reactants, transition state structures (TSs) and products and studied the effect of substituents on the N-aryl and the **2**-aryl aromatic ring on the energy of activation. The synchronicity of the process and the nature of non-covalent interactions were studied to gain insight into the reactivity and selectivity of these molecules. We examined the reactivity of **2**-arylhydrazone derivatives, which show reaction rates about three orders of magnitude slower than the parent β-ketoamide. We evaluated two competing mechanisms involving cyclic TSs of six and four members. This study included electronic descriptors, e.g., NBO analysis, IGM/IBSI, Wiberg bond indexes, and intrinsic reaction coordinate calculations (IRCs). We introduce a new descriptor, Dynamic Synchronicity, *SyD*, for mechanistic and kinetic characterization. To the best of our knowledge, this work is the first comprehensive theoretical study of the system ketoamide/aryl hydrazone. Multivariate statistical methods, i.e., principal component analysis (PCA) and hierarchical cluster analysis (HCA), are useful tools for the selection of the atoms involved in the TSs.

## 1. Introduction

**Scheme 1:**
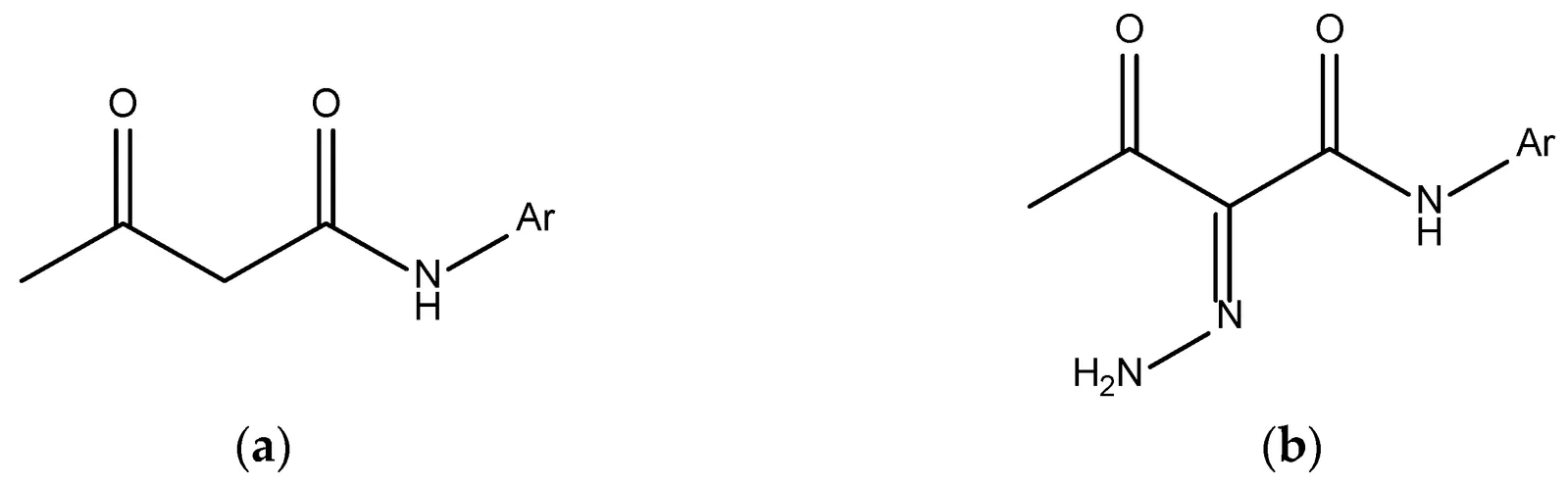
Structure of (**a**) N-aryl-**3**-oxobutanamide and (**b**) N-aryl-**2**-arylhydrazone-**3**-oxobutanamide.

N-aryl-3-oxobutanamides (β-ketoamides), Scheme 1, are versatile building blocks defined by an aryl group attached to the amide nitrogen and a ketone group at the beta position. They are widely used as intermediates in organic synthesis and pharmaceutical research.

In the industry, β-ketoamides have been used as precursors of aromatic isocyanates, which are intermediates in the synthesis of polyurethanes, surface coatings, adhesives, and polymer foams [1,2,3]. Thermal stability control and decomposition pathways of β-ketoamide precursors are critical for optimizing synthesis processes and the stability of final materials [4,5].

The structural motif β-ketoamide is found in the pharma industry [6], agrochemicals [7], and functional materials [8]. The study of thermal stability contributes to the understanding of degradation pathways, storage conditions, and industrial safety [9,10]. Ketoamides are model systems to study electronic and structural factors and the impact of the thermal stability of nitrogenated carbonyl compounds [11].

Arylhydrazone derivatives (Scheme 1b) possess a functional group present in medicinal chemistry [12,13], supramolecular chemistry [14], and molecular sensors [15,16]. Hydrazones have been used as units of molecular recognition, chemical switches, and synthesis precursors. Arylhydrazone derivative systems allow exploration of the impact of intermolecular interactions, stereoelectronic effects and electron density distribution on the stability and kinetics of reactions [14,17,18,19].

Gas-phase pyrolysis reactions are systems of interest because they are unimolecular processes, solvent-free and catalysis-free. These characteristics allow us to analyze the intrinsic reactivity of molecules, which depends only on the molecular structure, and study substituent effects without influences from the media or external agents [20,21,22].

**Scheme 2:**
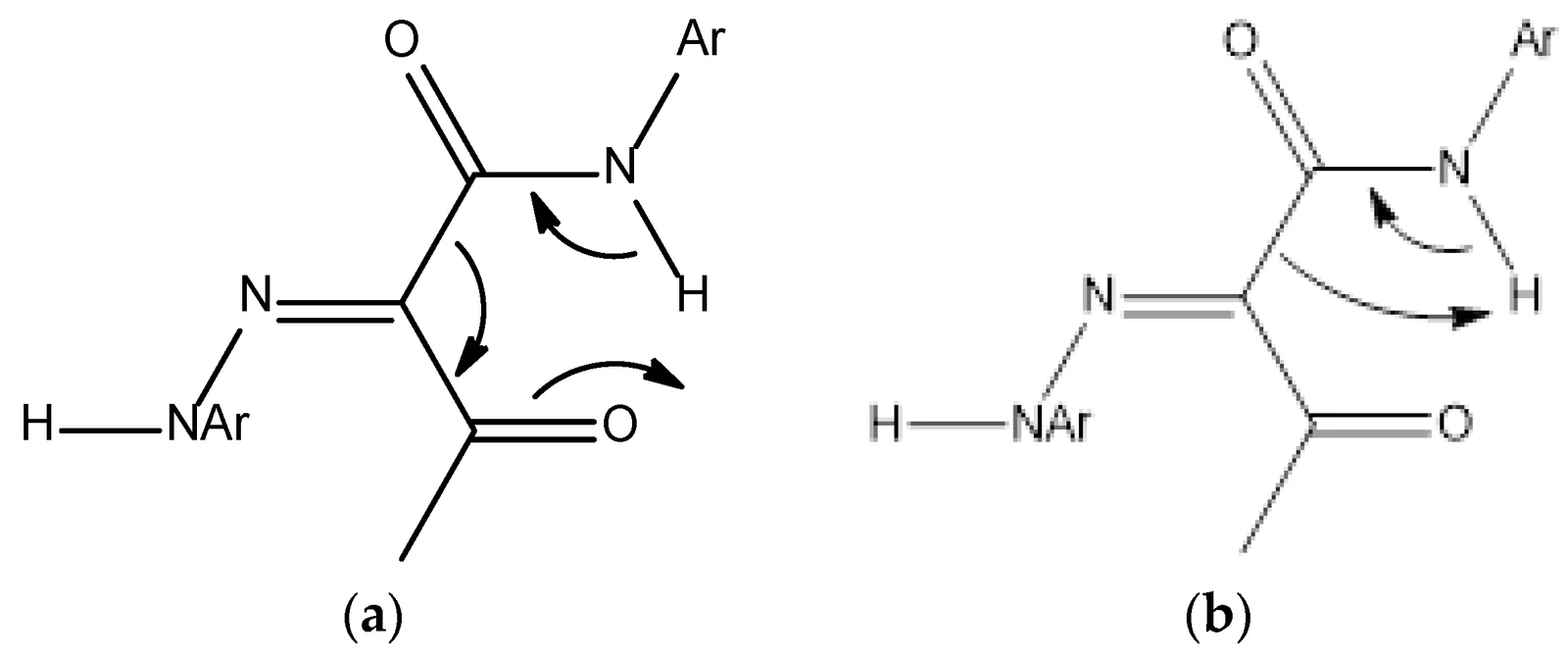
Alternative reaction pathways proposed for arylhydrazone derivatives: through a cyclic six-member TS (**a**), and competing reaction pathway through a cyclic four-member TS (**b**) [23].

The proposed mechanism for the thermal decomposition of β-ketoamides and thiones is an asynchronous concerted process. This mechanism proceeds through a cyclic six-member ring TS, depicted in Scheme 2a.

The proposed concerted asynchronous mechanism, illustrated in Scheme 2a, involves intramolecular proton transfer from the N-H group to the photophilic carbonyl oxygen (C=O), leading to molecular fragmentation [23]. The proton transfer occurs in a concerted asynchronous fashion. The fragmentation reaction forms an aryl isocyanate (O=C=N-Ar) and a ketone or its tautomer. The cyclic TS proposed is supported by previous theoretical studies in similar systems, and it is consistent with the typical kinetic parameters of polar unimolecular reactions [24,25,26,27]. Scheme 2 also provides a four-path mechanism to evaluate the competing reaction pathways. We carried out a comprehensive computational study to characterize the energetic profiles and electronic properties of the two competing reaction pathways.

**Scheme 3:**
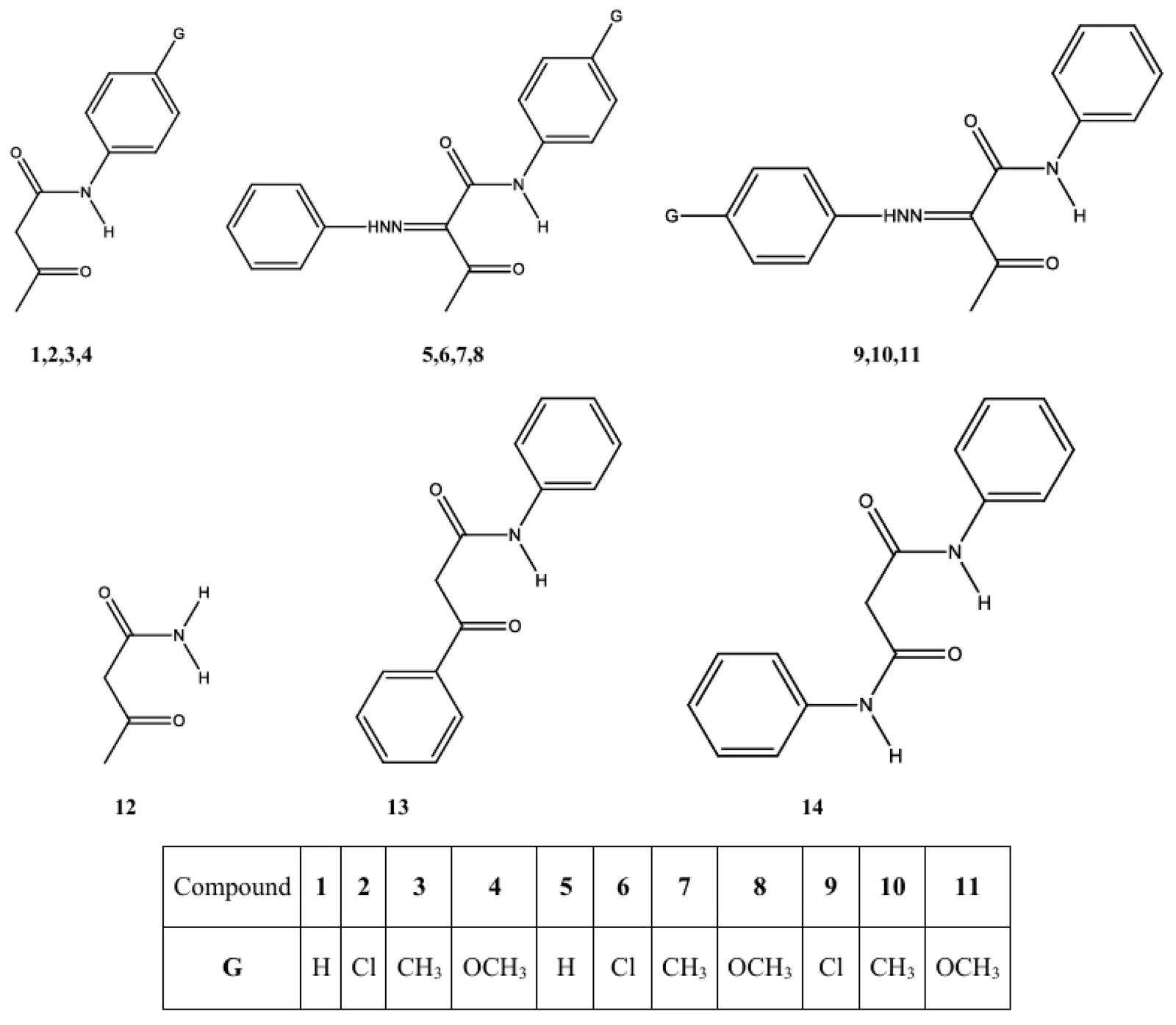
Compounds included in the experimental study by Malhas et al. [23].

An experimental work by Malhas et al. [23] studied the pyrolysis of fourteen N-aryl β-ketoamides and 2-arylhydrazo derivatives. The kinetic parameters reported (Table 1) show a great difference in reactivity: the arylhydrazone derivatives (compounds **5**–**11**) are over 1000 times less reactive than the parent ketoamides (compounds **1**–**4**, **12**–**14**), Scheme 3.

The Arrhenius parameter, logA, is related to the entropy of activation. Larger values of log A are associated with more positive entropies of activation, implying less ordered and higher conformational flexibility TSs.

In reactions with cyclic TSs mechanisms, the ring size impacts the entropy of activation ΔS^‡^. Small rings, e.g., four-member cyclic TSs are constrained, conformationally restricted, and associated with significantly negative ΔS^‡^ and low log A values, typically between 10 and 12. Larger cyclic TSs, e.g., six-member rings, are more flexible, have more rotational freedom and are associated with less negative ΔS^‡^, and consequently larger log A values, between 12 and 14.

Malhas et al. reported log A values of 12 for β-ketoamides and 13 for arylhydrazones, consistent with a cyclic six-member TS mechanism. The difference suggests that the TS for arylhydrazone thermal decomposition is more flexible, less ordered than the TS for β-ketoamides.

To explain the difference, Malhas et al. suggested that for arylhydrazone derivatives, both the hydrazone nitrogen (N-H) and the amide hydrogen could interact with the protophilic oxygen of the carbonyl group. To the best of our knowledge, the proposed mechanisms have not been validated by comprehensive theoretical studies, and the factors that govern this competition, e.g., electronic and stereoelectronic factors, the relative acidity of the proton in the amide N-H and hydrazone groups, the protophilicity of the carbonyl oxygen, the polarity of bonds, and the role of non-covalent intramolecular interactions, have not been described.

Density functional theory (DFT) methods have been consolidated as an important tool to study gas-phase reaction mechanisms, allowing the characterization of TSs, calculation of activation energies and evaluation of electronic effects, with adequate precision and low computational costs [28,29]. DFT studies of unimolecular pyrolysis have been shown to reproduce experimental kinetic parameters, allowing the validation of proposed mechanisms [30,31].

Previous computational works studied the thermal decomposition of systems with similar structural motifs, e.g., β-ketoesters [32], thionamides [33], and other functionalized amides [34]. These works proposed cyclic six-membered TSs for the thermal decomposition and highlight the role of an intramolecular proton transfer in the C-N bond breaking. The methods include range-separated DFT calculations [35].

Natural bond orbital (NBO) methods have been used extensively to quantify the electronic redistribution and donor–acceptor interactions in TSs of elimination reactions [36]. Wiberg bond indexes provide effective bond orders and have been used to evaluate the advance in the reaction coordinates and synchronicity of concerted reactions [37].

In this work, we use principal component analysis (PCA) to determine the stereoelectronic factors in the gas-phase pyrolysis of N-aryl-3-oxobutanamides (β-ketoamides) and the 2-arylhydrazone derivatives. We propose the utilization of the synchronicity parameter along the reaction coordinate, from reactants to products, introducing a new descriptor, Dynamic Synchronicity, *SyD*.

In addition, we used the independent gradient model (IGM) to visualize and characterize non-covalent interactions that can contribute to stabilizing or destabilizing reactive geometries [38].

The computational protocol in this work includes optimization of geometries with DFT functionals with dispersion corrections, energy calculations with extended basis sets, NBO analysis, and IGM and IRC calculations. This methodology has been used in similar mechanistic studies [39]. This study aims at validating (or disproving) the TS proposed in the experimental study by Malhas et al., and the quantification of electronic and stereoelectronic factors which contribute to the observed difference in reactivity between β-ketoamides and arylhydrazone derivatives.

## 2. Results and Discussion

Table 1 displays the experimental data reported by Malhas et al. for the gas-phase pyrolysis at 450 K. All reactions showed first-order kinetics. Arrhenius parameters log A (s^−1^) and Ea (kJ mol^−1^) are in the expected range for unimolecular gas-phase thermal elimination [40,41,42,43,44,45,46,47,48,49,50,51,52,53]. The fragmentation reaction forms an aryl isocyanate (O=C=N-Ar) and a ketone or its tautomer. Ketenes (RCH=C=O) were not observed in the pyrolysis products. The two mechanisms, shown in Scheme 2, explain the formation of aryl isocyanate, Ar-N=C=O; i.e., the product formation can be explained by a concerted mechanism through a six-member cyclic TS, 2a, and also by an alternate pathway through a cyclic four-membered TS, 2b. In both proposed mechanisms, the bond between the amide carbonyl and the alpha carbon breaks, and an aryl isocyanate forms, Ar-N=C=O. In mechanism 2a, Scheme 2, the cyclic six-member TS is thermodynamically more stable. In this pathway, a proton transfer occurs from the amide nitrogen, N-H, to a protophilic center, e.g., the oxygen atom in the carbonyl group, C=O. In mechanism 2b, involving a cyclic four-member TS, the amide proton N-H is transferred to the alpha carbon. This is less favorable since carbon is less protophilic than the β-carbonyl oxygen. We discuss the thermodynamic and kinetic data of mechanism 2a, evaluating the possibility of mechanism 2b.

Data collected in Table 1 show that **2**-arylhydrazone-substituted β-keto anilides (N-aryl-β-ketoamides), structures 5–8, are less reactive than the non-substituted analogues (1–4) by a factor of ≥1.4 × 10^3^.

### 2.1. Computational Study of β-Ketoanilides, Compounds ***1**–**4***

**Figure 1:**
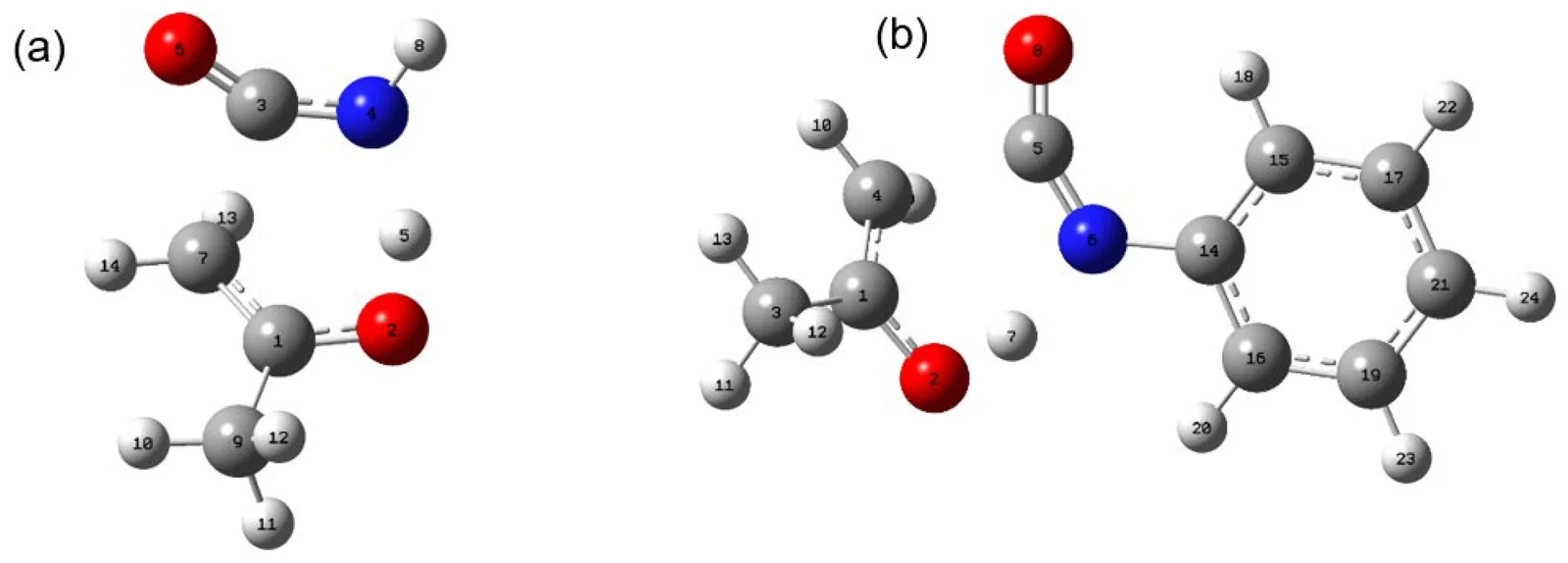
Computational models of the TS for acetoacetamide (**a**) and N-phenylacetoacetamide (N-Phenyl-3-oxobutanamide) (**b**), at B97D-GD3BJ/Def2TZVP, 450 K.

Table 2 and Table 3 display thermodynamic parameters calculated using the B97D-GD3BJ/Def2TZVP level of theory at 450 K. We used acetoacetamide (compound **12**, Figure 1) as a reference for the discussion of reactivity differences. The calculated energy of activation for acetoacetamide 129.87 kJ/mol, obtained by assuming the mechanism depicted in Scheme 2a, is comparable with the experimental value of 121.3–127.3 kJ/mol with experimental error. This indicates that the mechanism proceeds through a six-member TS. The calculated energy of activation for *N*-phenyl-3-oxobutanamide (*N*-phenyl-acetoacetamide), compound **1**, Figure 1, Table 2, is slightly lower than that of acetoacetamide. The Ea decreases from 129.87 to 126.67 kJ/mol, suggesting that the phenyl group stabilizes the six-member TS, facilitating the reaction., i.e., the reaction is faster. Substitution in the aromatic ring para position by chlorine (p-Cl, compound **2**), methyl (p-Me, compound **3**), and methoxy (p-OMe, compound **4**) does not make significant changes; calculated energies of activation are around 126 kJ/mol.

Higher reactivity cannot be explained solely in terms of differences in energy of activation. The free energy of activation, ΔG, is a better parameter to describe reactivity, as it contains entropy changes, included in the experimental log A. For the p-chloro substituted compound **2**, log A is 14, implying more favourable entropy of activation, contributing to the faster rate. The calculated entropy of activation is −6.69 J/Kmol, the smallest negative value in the series. The slowest reaction is observed for compound **3**, with a p-methyl substituent, and log A 9.75, indicating a very organized, restricted TS, with a calculated entropy of activation of −9.20 J/Kmol. The small differences in the calculated entropy of activation between these compounds suggest the functional DFT (B97D-GD3BJ/Def2TZVP underestimates entropic effects or the balance between electronic and steric effects in the TS.

### 2.2. Computational Study of Phenylhydrazone Derivatives of β-Ketoanilides, ***5**–**11***

**Scheme 4:**
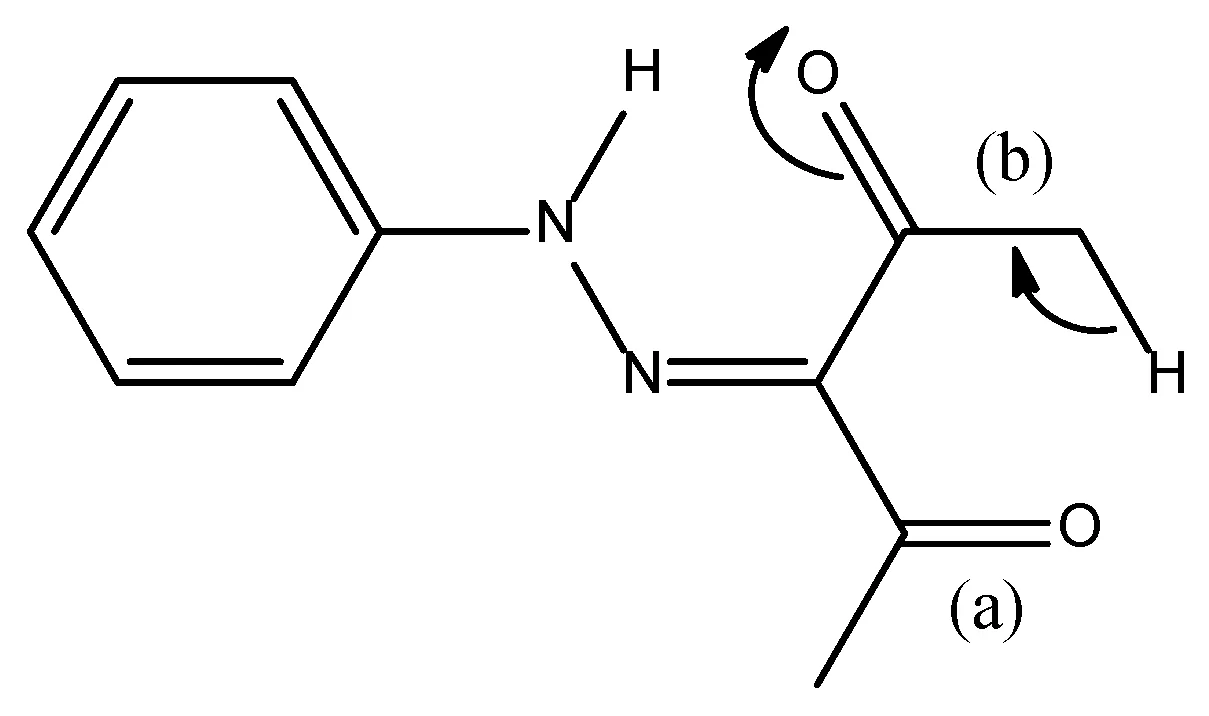
Assistance of the phenylhydrazone group in the elimination reaction. The effect increases the positive charge of the alpha hydrogen next to the carbonyl (b), facilitating the transfer of the proton, which is transferred to the oxygen in the carbonyl group (a).

The influence of arylhydrazone substituents on the reactivity of acetoacetamide and acetanilides (**1**–**4**), compared to non-substituted, is significant, as observed in the experimental data and in theoretical calculations. Analysis of the arylhydrazone effect on the kinetics and mechanism of these systems provides an opportunity to study structure–reactivity relationships, Scheme 4.

The phenylhydrazone group on carbonyl (b) helps increase the positive charge of the hydrogen adjacent (α to carbonyl b), facilitating the proton transfer to the oxygen of the carbonyl group (a), (Scheme 4). The effect is an increase in the pyrolysis rate compared to acetoacetamide, due to the assistance of the phenylhydrazone group.

**Figure 2:**
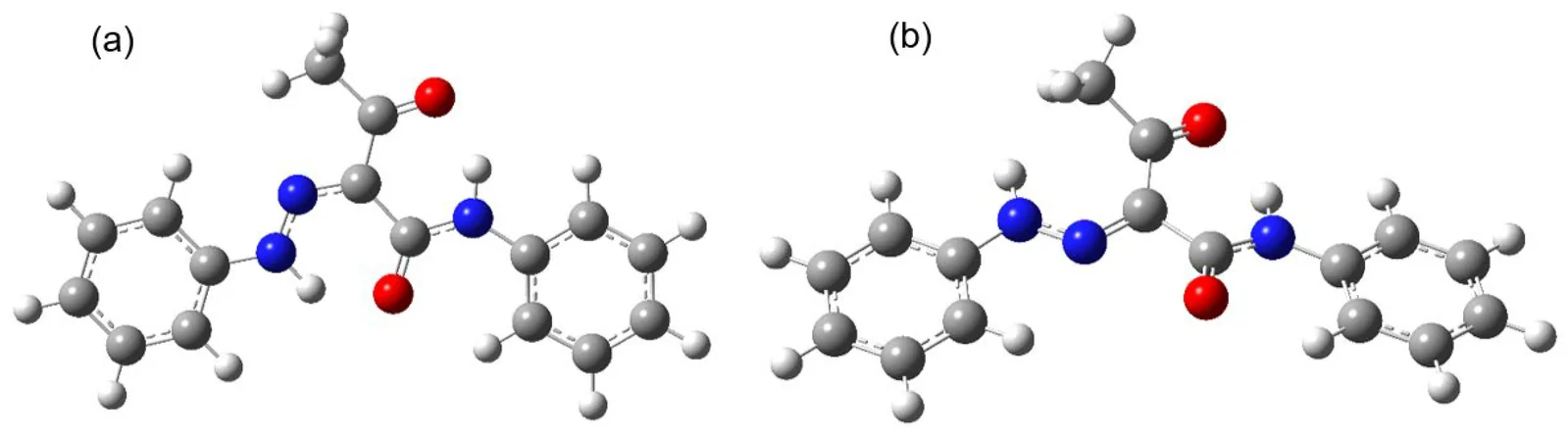
Conformers of compound **5**. (**a**) Conformer A: The hydrazone N-H hydrogen interacts with the carbonyl oxygen. This conformation does not lead to the observed fragmentation products. (**b**) Conformer B: The amide N-H hydrogen orients toward the carbonyl oxygen. This is the reactive conformation leading to the observed products. Energy difference: ΔG = −10 to −12 kJ/mol at 450 K (B is more stable).

An interaction between the keto group (C=O) and the hydrogen of the arylhydrazone group (N-H) in a conformation of compound **5** is depicted in Figure 2a. This interaction is an alternate pathway, affecting the rate of the pyrolysis reaction. This pathway, however, does not produce the observed fragmentation products. Figure 2b shows the reactant conformation leading to product formation, used in this work. An interaction between the keto group (C=O) and the hydrogen of the arylhydrazone group (N-H) in a conformation of compound **5** is depicted in Figure 2a. This interaction is an alternate pathway, affecting the rate of the pyrolysis reaction. This pathway, however, does not produce the observed fragmentation products. Figure 2b shows the reactant conformation leading to product formation, used in this work.

Table 2 and Table 3 show the influence of the arylhydrazone group, according to the six-member TS mechanism and four-member TS mechanism, depicted in Scheme 2. Results show that the mechanism through a four-member TS gave larger values of ΔG^‡^ and Ea than the six-member TS; thus, the four-member TS is less favourable. In the case of the six-member TS from compound **14**, the conformation shown in Figure 2b gave a calculated ΔG^‡^ over 200 kJ/mol. Rotation of the phenylhydrazone group to the conformation shown in Figure 2b produced a ΔG^‡^ value in agreement with the experimental value.

**Figure 3:**
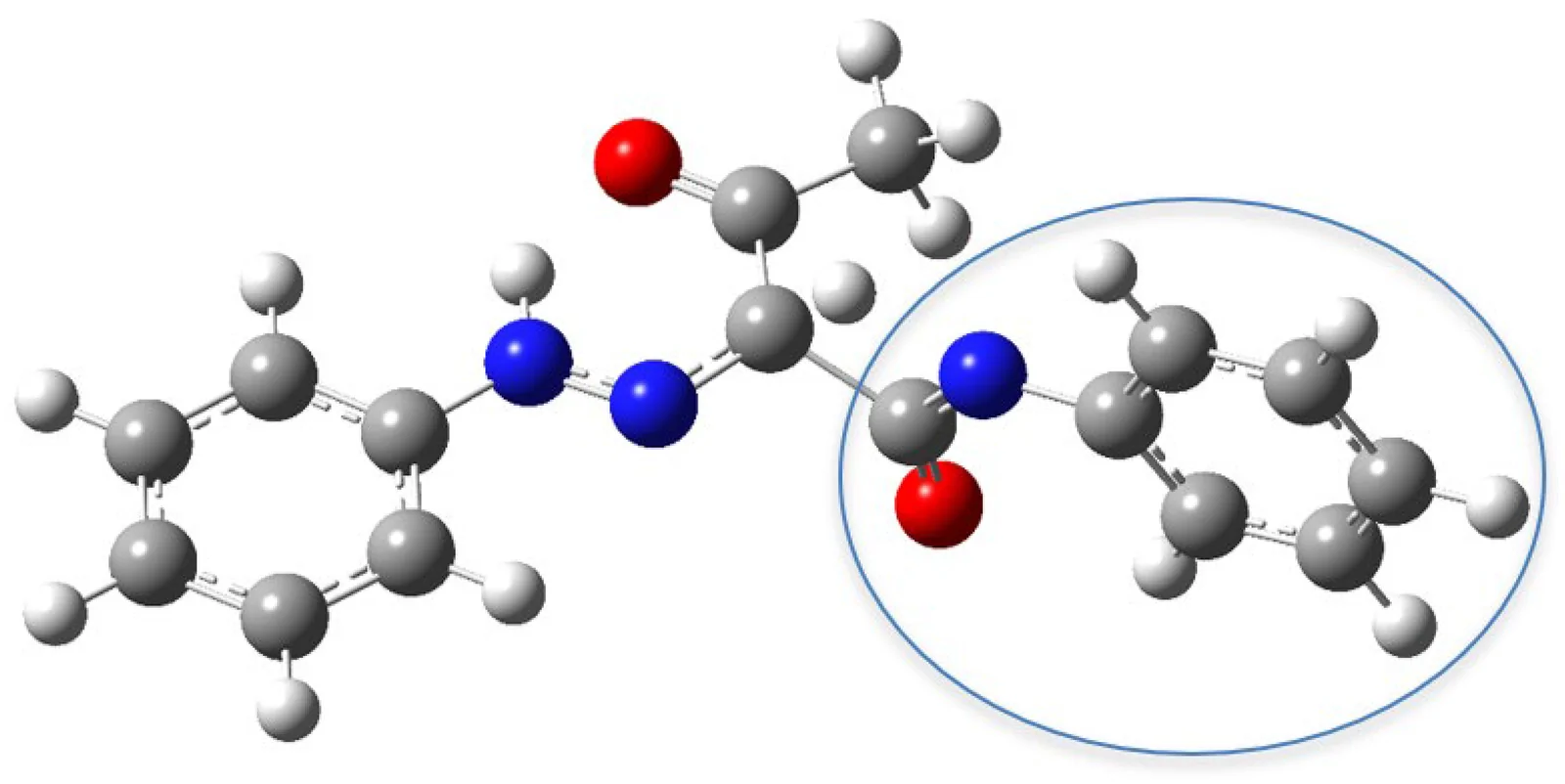
Four-member cyclic TS for compounds **5**–**11**, optimized at the B97D-GD3BJ/Def2TZVP level of theory at 450 K. To enable the migration of the amide N–H hydrogen toward the α-carbon, the fragment highlighted in the blue circle undergoes a ~90° rotation around the C–C bond. The C–C bond that breaks during the reaction is oriented perpendicularly to the plane of the migrating hydrogen, facilitating the fragmentation process.

The four-member TS structure was also used in this study for compounds **5**–**11**, and it is depicted in Figure 3. In this TS, the amide N–H hydrogen migrates toward the α-carbon atom. To enable this migration, the group highlighted in the blue circle rotates approximately 90° around the C–C bond, orienting the α-carbon favorably for the hydrogen transfer. The hydrazone N–H hydrogen remains in proximity to the oxygen of the acetyl group, as shown in the figure. This conformational rearrangement is essential to achieve the four-member cyclic geometry and allows the subsequent C–C bond cleavage.

**Figure 4:**
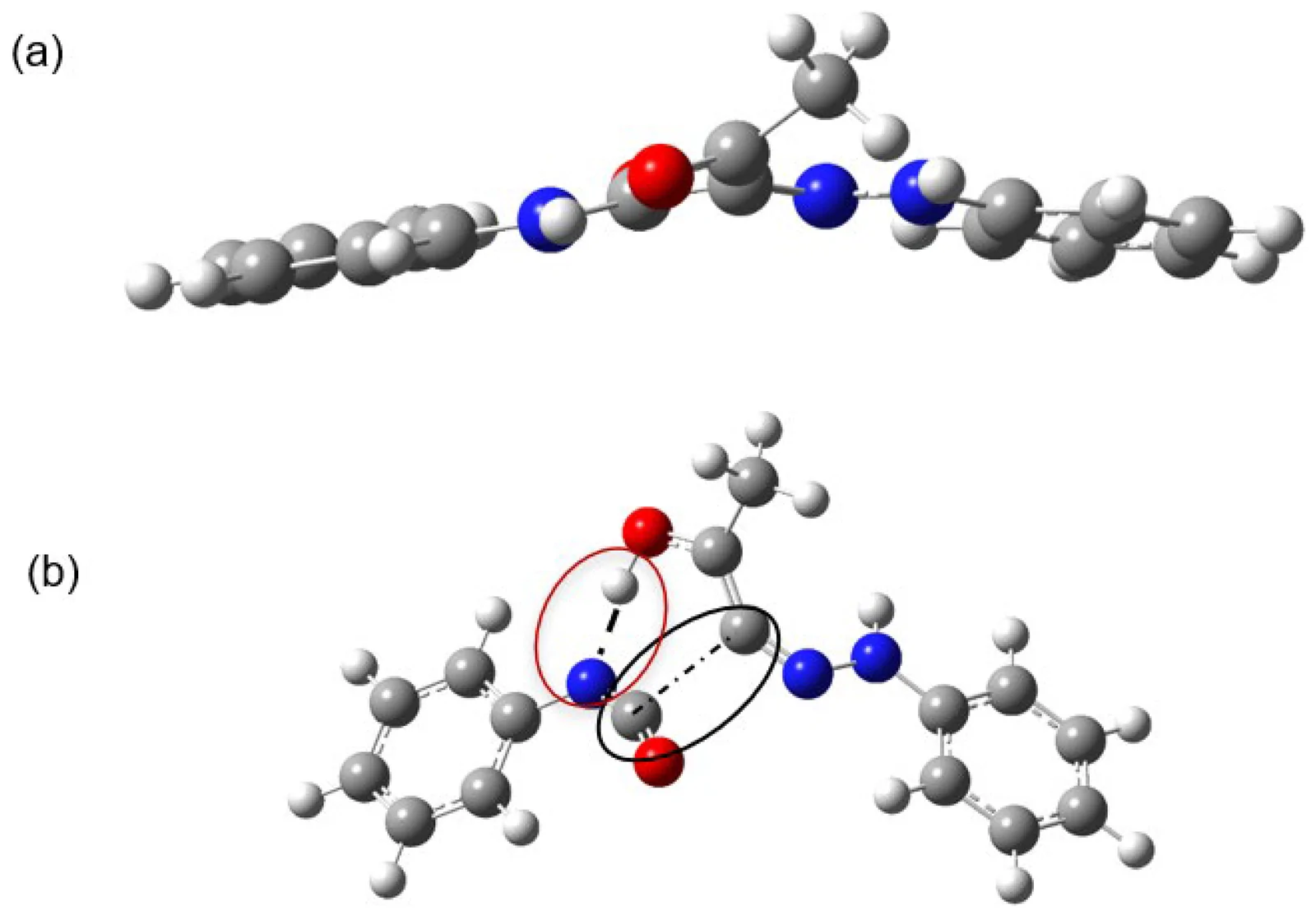
(**a**) Top depicts another view of the conformer in Figure 2b, showing an almost planar structure except for the methyl group, clearly off plane. (**b**) Bottom depicts the six-member TS structure found for compounds **5**–**11**, from GD3BJ/Def2TZVP calculations at 450 K.

Figure 4 depicts another view of Figure 2b. The aromatic rings and the center of reaction are approximately in one plane, and the methyl group is in the plane. The TS in Figure 4b shows early hydrogen migration, and the C-C bond is significantly broken. The energies of activation, Ea and enthalpies of activation of aryhydrazone (compounds **5**–**11**) are much higher than the Ea of the parent ketoanilides (compounds **1**–**4**), which explains the lower reactivity observed experimentally. For compounds **5**–**8**, with substituents -H, -Cl, -CH_3_, and -OCH_3_ in the para position of the anilide phenyl ring, small changes in Ea are observed, 177–180 kJ/mol for the six-member TS mechanism, suggesting the substituent effect is negligible on the energy barrier. By contrast, for compounds **9**–**11**, where the substituent is at the para position of the aryhydrazone ring, the effect is significant. Compound **11** (p-OCH_3_) has the lowest Ea (~177 kJ/mol), followed by compound **10** (p-CH_3_), while compound **9** (p-Cl) has the highest Ea (~180 kJ/mol).

These results imply that electron-donor groups (EDGs) (p-OCH_3_ and p-CH_3_) on the arylhydrazone group stabilize the six-member cyclic TS, while the electron-withdrawing group (EWG), p-Cl, destabilizes the TS. Comparing the mechanisms, the six-member cyclic TS is favored over the four-member cyclic TS by approximately 10–12 kJ/mol, which confirms the proposal by Malhas et al. Entropies of activation (ΔS) for compounds **5**–**11** are positive (25–30 J/Kmol) for the six-member TS mechanism, indicating a less organized TS configuration, while for the four-member TS mechanism, the entropy change is zero or slightly positive, implying a more restricted TS configuration.

Regarding the functional/basis set performance, B97D-GD3BJ/Def2TZVP calculation reproduces the observed reactivity trend; that is, aryhydrazone derivatives **5**–**11** have barriers significantly higher (~178 kJ/mol) than ketoanilides **1**–**4** (~126 kJ/mol), in agreement with experimental results (~158–164 kJ/mol for compounds **5**–**11** vs. ~105–136 kJ/mol for compounds **1**–**4**). However, calculations systematically overestimate the Ea by 15–20 kJ/mol for compounds **5**–**11**. This discrepancy suggests that even though the functional captures the principal electronic effects, it underestimates the stabilization of the TS by non-covalent interactions, or finer electron correlation effects, especially in aryhydrazone derivatives with extended conjugation. Despite the systematic error, the calculation reproduces the experimental reactivity trend and substituent effect; thus, the level of theory is robust enough to establish relative reactivity and validate the predominant mechanism as a cyclic six-member TS.

### 2.3. HOMO–LUMO, IBSI, and NPA

The optimized geometries of the 14 compounds obtained from DFT calculations using the B97D-GD3BJ functional with the Def2TZVP basis set were verified by vibrational analysis to confirm that the structures are true local minimums on the potential energy surface; that is, they lack imaginary frequencies. We constructed nine matrices from the compounds’ descriptors; for the compounds, we were able to obtain an IRC. The IRC gave 201 points for each compound.

The energy gap between frontier orbitals, HOMO and LUMO, has been used to characterize chemical reactivity and stability. However, within conceptual DFT (CDFT), the fundamental descriptor is hardness (η = (I − A)/2 ≈ (ELUMO − EHOMO)/2), which measures resistance to electron cloud polarization. For completeness, we report both the HOMO–LUMO gap, ΔEg, and the hardness η. Table 4 shows the HOMO–LUMO energies and the energy gap, ΔEg, for the molecules in this study.

Calculated global descriptors, e.g., chemical potential, ionization energy, hardness, and softness, are presented in Table S2. Ionization energy is a fundamental descriptor for chemical reactivity. The high energy of ionization implies chemical stability. Hardness and softness descriptors are also used to describe molecular stability and reactivity. The hardness descriptor illustrates resistance to deformation or polarization of the electron cloud in atoms, ions and molecules under a perturbation. Molecular descriptors were calculated from the energies of frontier orbitals, HOMO–LUMO, using the DFT/B97D-GD3BJ/Def2TZVP level of theory, in the gas phase.

Analysis of the energy gap HOMO–LUMO, ΔEg, in Figure S1 revealed that ΔEg decreases from ~3.75 eV in the reactants (point 1 of the IRC, M1) up to ~3.57 eV in the TS region (M93-M96), and increases again to ~3.95 eV in the products region (M201). The point of maximum gap, ΔEg of 3.6148 eV (HOMO −5.9471 eV and LUMO −2.3323 eV), occurs at the TS (M101), indicating greater polarizability and less kinetic stability, to facilitate the charge transfer implied in bond breaking and formation. Global descriptors derived from HOMO–LUMO energies at M101 are hardness (η) = 1.8074 eV, chemical potential (μ) = −4.1397 eV, softness (S) = 0.5533 eV^−1^, electrophilicity index (ω) = 4.7408 eV and maximum charge transferred ΔNmax = 2.2904 e. These values confirm that TS is softer, more polarized, has a greater capacity to accept electrons, and is more reactive at this point on the potential energy surface.

The intrinsic bond strength index (IBSI), derived from the independent gradient model (IGM), is a powerful method to measure and compare the strengths of chemical bonds. IBSI and electron density (ρ) analysis along the IRC quantifies electronic exchange between fragments participating in the reaction. At the TS (M101), IBSI associated with N4-H4 bond breaking decreases significantly, to 0.316, while IBSI associated with O2-H5 bond formation increases to 0.912, illustrating the asynchronous character of the six-member TS mechanism. Electron density (ρ) analysis in the TS shows values of ρO2−H5 = 0.245 and ρN4−H5 = 0.096, indicating a hydrogen bond N4–H5···O2, stabilizing the TS, Figure S2.

Figure S3 shows atomic charges from natural population analysis (NPA). The hydrogen atom in the amide group, H4, becomes more positive, qH5 = +0.47149 e, consistent with the proton transfer to the oxygen of the carbonyl group, O2, which increases its charge to qH5 = +0.47149 e, from its value in the reactant, −0.53942 e. The amide nitrogen, N4, shows a decrease in charge to a value qN4 = −0.85727 e, compared to the reactant, with a value of −0.78444 e, illustrating the breaking of the bond N-H, with electron delocalization toward the aromatic ring. Carbon atoms C1, C3, and C7 also show significant changes: C1 becomes more positive (qC1 = +0.45863 e in the TS vs. +0.57194 e in the reactant), C3 becomes more positive (qC3 = +0.73602 e in the TS vs.+0.62233 e in the reactant), and C7 becomes more negative (qC7 = −0.59461 e in the TS vs.−0.60166 e in the reactant), illustrating the charge redistribution associated with C3-C7 bond breaking and product formation.

Jointly, these analyses, HOMO–LUMO gap, ΔEg, intrinsic bond strength index, IBSI, and atomic charges from natural population analysis, NPA, provide a consistent, multifaceted image of the pyrolysis process. Reactivity is controlled by the capacity of the system to polarize the electron cloud and facilitate the charge redistribution in the TS. This ability is modulated by electronic effects of the substituents at the aromatic ring, with differences in the behavior between ketoanilides (**1**–**4**) and the arylhydrazone derivatives (**5**–**11**).

### 2.4. Principal Component Analysis (PCA)

For PCA, due to the amount of data involved, we used the IRC information of the atoms involved in the six-member TS, Table 4. We built a 201 × 30 PCA matrix using 30 variables: HOMO (eV), LUMO (eV), gap (eV), I, A, χ, η, μ, S, ω, ΔN, IBSI_O2-H5, IBSI_H5-N4, IBSI_N4-C3, IBSI_C3-C7, IBSI_C7-C1, IBSI_C1-O2, electron density ρ_O2-H5, ρ_H5-N4, ρ_N4-C3, ρ_C3-C7, ρ_C7-C1, ρ_C1-O2, C1, and charges qC3, qC7, qN4, qH5, and qO2.

For compound **12**, data was auto-scaled to ensure all variables were comparable on the same scale. The objective of the PCA was to reduce the dimensionality of the data, to identify the most relevant variables and determine which variables can explain the chemical reactivity of the compounds in this study. Table 4 also shows the variables included in PC1 calculated for each IRC point for compound **12**. Variance analysis revealed that the three principal components, PC1, PC2, and PC3, explain 99.31% of the total variance, with PC1 87.32%, PC2 10.46% and PC3 1.54%. We used these components for the analysis to interpret the chemical reactivity of the compounds; the inclusion of more components is more likely to introduce noise than give significant information.

**Figure 5:**
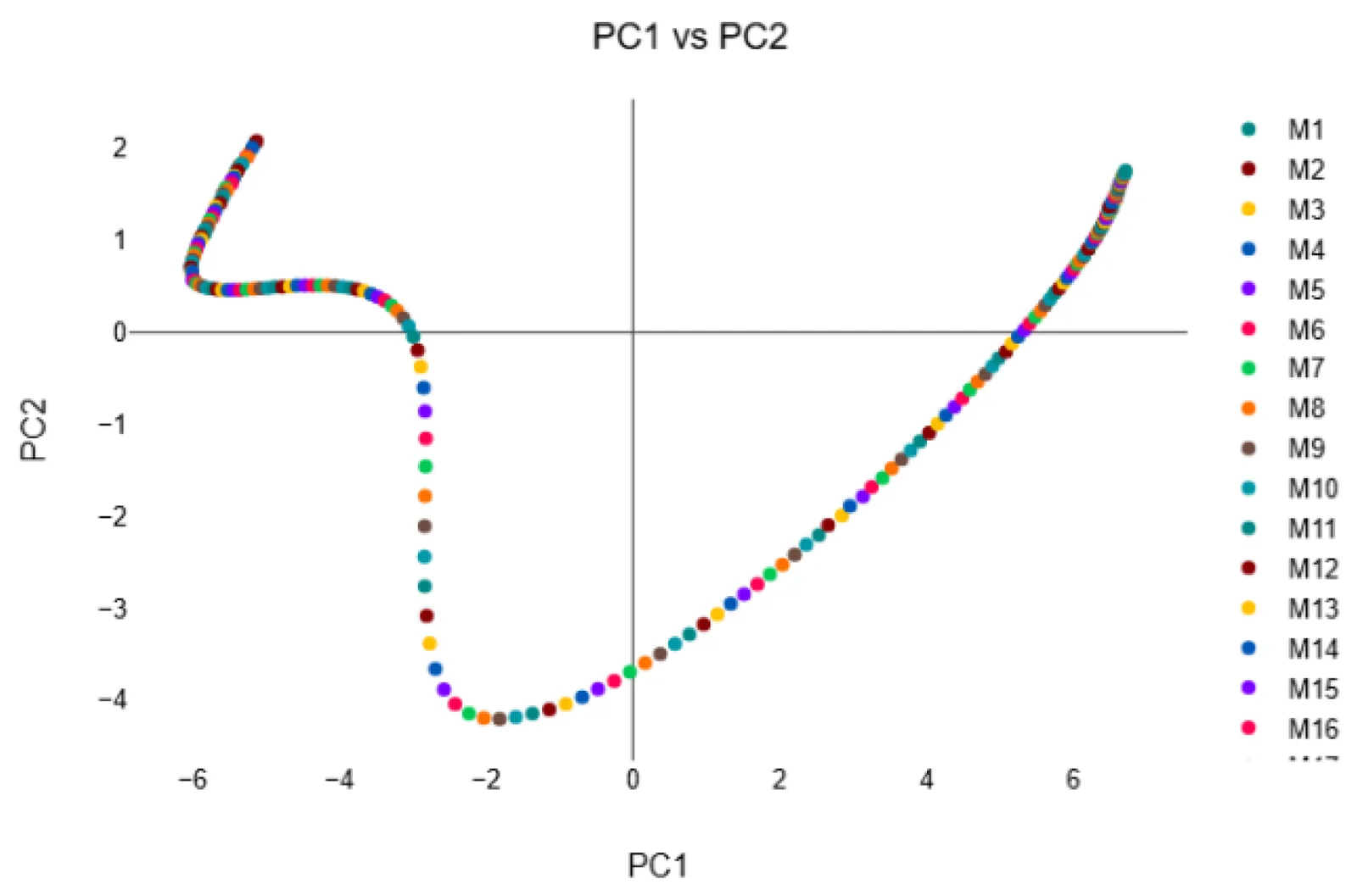
Plot of principal component PC1 vs. PC2 values of 201 points of the intrinsic reaction coordinate (IRC), for the pyrolysis of acetoacetamide (compound **12**), calculated at B97D-GD3BJ/Def2TZVP level of theory, at 450 K. Structures with higher reactivity are characterized by PC1 > 0; less reactive structures have PC1 < 0.

A plot of the distribution of scores PC1 vs. PC2 in the structures along the IRC (M1, M2, etc.) is shown in Figure 5. We observe two main groups. The most reactive structures have larger positive PC1 values, while less reactive structures have negative PC1 values. This information indicates that the PC1 value is critical for reactivity, while PC2 provides a secondary classification associated with fine-structural effects or electron-density variations.

The charge vector, a.k.a., the loading vector, represents the weights or coefficients assigned to each original variable to construct a specific principal component. The charge vector for PC1 is expressed by a linear equation with coefficients for the weight of the variables. The vector is as follows:

PC1 = 0.92[HOMO] + 0.94[LUMO] + 0.89[Gap ΔEg] − 0.92[I] − 0.94[A] − 0.93[χ] + 0.89[η] + 0.93[μ] − 0.88[S] − 0.94[ω] − 0.94[ΔN] − 0.95[ΔEn] − 0.94[ΔEe] + 0.97[IBSI_O2-H5] − 0.95[IBSI_H5-N4] + 0.99[IBSI_N4-C3] − 0.95[IBSI_C3-C7] + 0.98[IBSI_C7-C1] − 0.98[IBSI_C1-O2] + 0.97[ρ_O2-H5] − 0.95[ρ_H5-N4] + 0.98[ρ_N4-C3] − 0.94[ρ_C3-C7] + 0.96[ρ_C7-C1] − 0.98[ρ_C1-O2] − 1.00[qC1] + 0.97[qC7] + 0.97[qC3] − 0.32[qN4] + 0.94[qH5] − 0.98[qO2]

From this equation, we selected the variables with positive coefficients, associated with higher chemical reactivity (PC1 > 0). The variables with greater weight in the PC1 charge vector comprise HOMO, LUMO, gap ΔEg (HOMO–LUMO gap), η (hardness), μ (chemical potential), bond strength: IBSI_O2-H5, IBSI_N4-C3, and IBSI_C7-C1, bond electron densities: ρ_O2-H5, ρ_N4-C3, and ρ_C7-C1, and atomic charges qC7, qC3 and qH5. These variables reflect the capacity of the compound to polarize the electron cloud, stabilize the TS by non-covalent interactions, and facilitate charge transfer along the reaction coordinate. The simplified charge PC1 vector is as follows:

PC1 = 0.92[HOMO] + 0.94[LUMO] + 0.89[Gap ΔEg] + 0.89[η] + 0.93[μ] + 0.97[IBSI_O2-H5] + 0.99[IBSI_N4-C3] + 0.98[IBSI_C7-C1] + 0.97[ρ_O2-H5] + 0.98[ρ_N4-C3] + 0.96[ρ_C7-C1] + 0.97[qC7] + 0.97[qC3] + 0.94[qH5].

**Figure 6:**
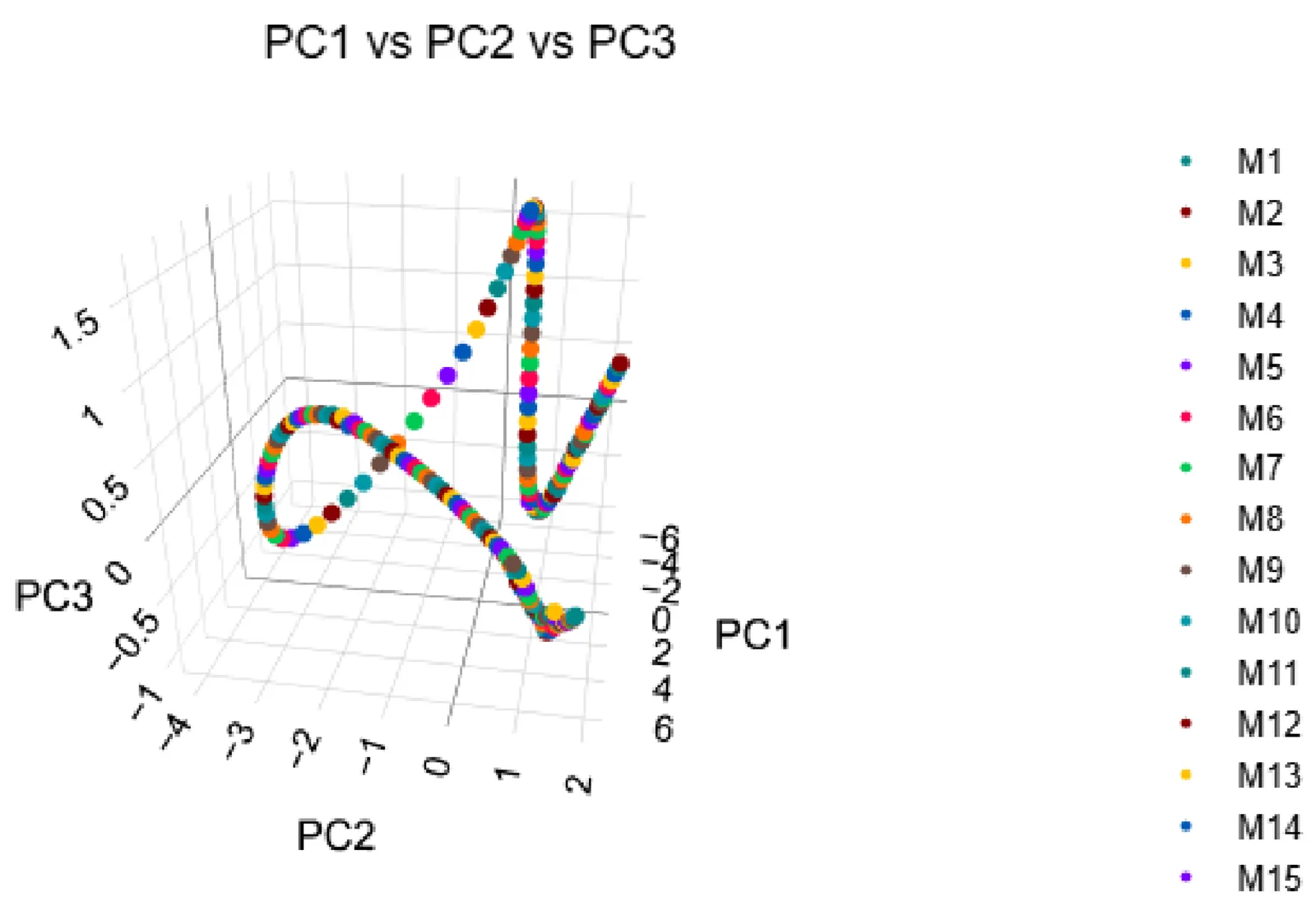
Three-dimensional plot of principal components PC1, PC2, and PC3 for 201 points in the IRC of compound **12**, calculated at B97D-GD3BJ/Def2TZVP level of theory at 450 K. The separation between reactive and non-reactive structures is observed along the PC1 axis, while PC2 and PC3 give complementary information.

Figure 6 shows a 3D plot of PC1, PC2, and PC3 values at each point of the IRC. The axes PC2 and PC3 give complementary information to the reactivity classification obtained with PC1. This 3D representation allows grouping and gives possible subclasses among reactive structures, which may be related to orientation in space or charge distribution in the TS. A fundamental aspect in Table 5 is the sign change in PC1 coefficients, from one compound to another, indicating an inversion in the contribution of some variables on the reactivity. For example, for compounds Cp12, Cp13 and Cp1-H, the coefficients of the variables HOMO, LUMO, ΔEg (HOMO–LUMO gap), η, μ, IBSI and atomic charges qC7, qC3, and qH5 are positive and consistent, indicating that the high contribution of these variables is associated with greater reactivity of the compounds, PC1 > 0. By contrast, for compounds Cp1-Cl, Cp1-Me, and Cp13-Cl, the coefficients of the variables HOMO, ΔEg (HOMO–LUMO gap), η, μ and charges qC7, qC3, and qH5 change sign (negative values), revealing the relationship between these variables and lower reactivity, PC1 < 0.

The change in sign shows that the nature of substituents, electron-donor groups (EDG), or electron-withdrawing groups (EWGs) changes the role of the descriptor in the reactivity of the compounds. For example, for compounds with chlorine (EWG), e.g., Cp1-Cl and Cp13-Cl, the variables hardness (η) and chemical potential (μ) have negative coefficients, implying that these compounds respond in an opposite way to the compounds with no substituent. For compounds that have positive sign coefficients for all variables, e.g., Cp12, Cp13, and Cp1-H, the reactivity is dominated by the polarization and charge transfer ability, while for the compounds with negative coefficients, e.g., Cp1-Cl, Cp1-Me, and Cp13-Cl, electronic and steric effects compete and modify the relative contribution (coefficient) of the descriptors.

**Figure 7:**
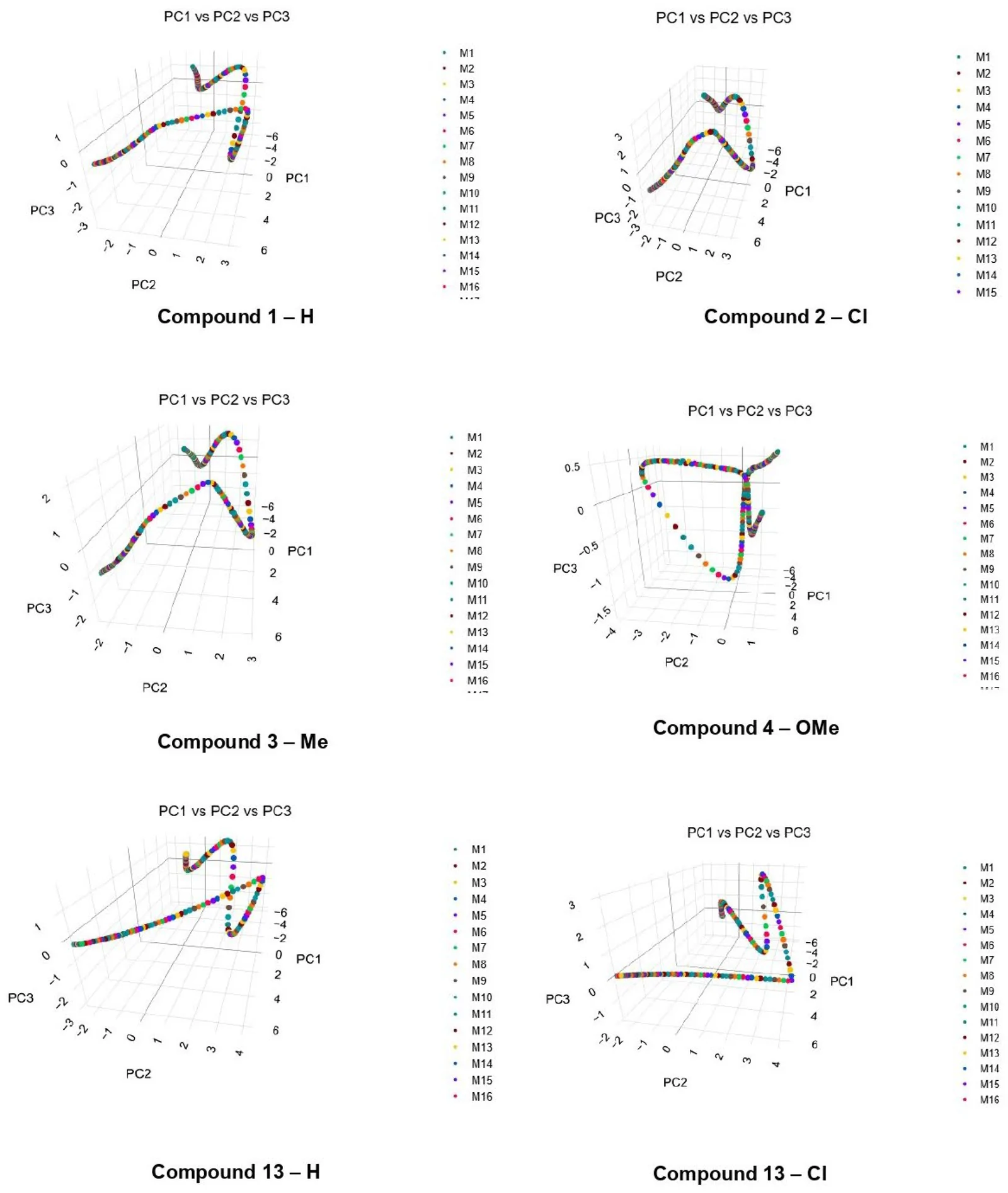
Three-dimensional graphical representation of principal components PC1, PC2, and PC3 and IRC points for the compounds studied. Compounds included possess substituents EDG (-Me, -OMe) and EWG (-Cl).

Figure S4 and Figure 7 are important to understand how the substituents modulate the reactivity along the reaction coordinate. Figure 4 (PC1 vs. PC2) displays the distribution of the 201 points in the IRC in 2D, where a clear separation is observed between the most reactive structures (PC1 > 0) and less reactive ones (PC1 < 0). This separation is relevant because PC1 represents a universal reactivity criterion, independent of the compound. Figure 7 is the 3D display of PC1 vs. PC2 vs. PC3, adding an additional layer of information that allows the visualization of the effect of EDG and EWG in the shape and orientation of the curve in the principal component space. The changes in the point distribution reflect profound changes in the electronic dynamic of the reaction, which manifest in the relative position of the TS and the dispersion of the points representing reactants and products. Figure S4 and Figure 7 demonstrate that PCA not only reduces the data dimensionality but also allows the identification of reactivity patterns that are not evident using individual descriptors. This study confirms PCA as an important tool for mechanistic studies.

### 2.5. Hierarchical Cluster Analysis (HCA)

**Figure 8:**
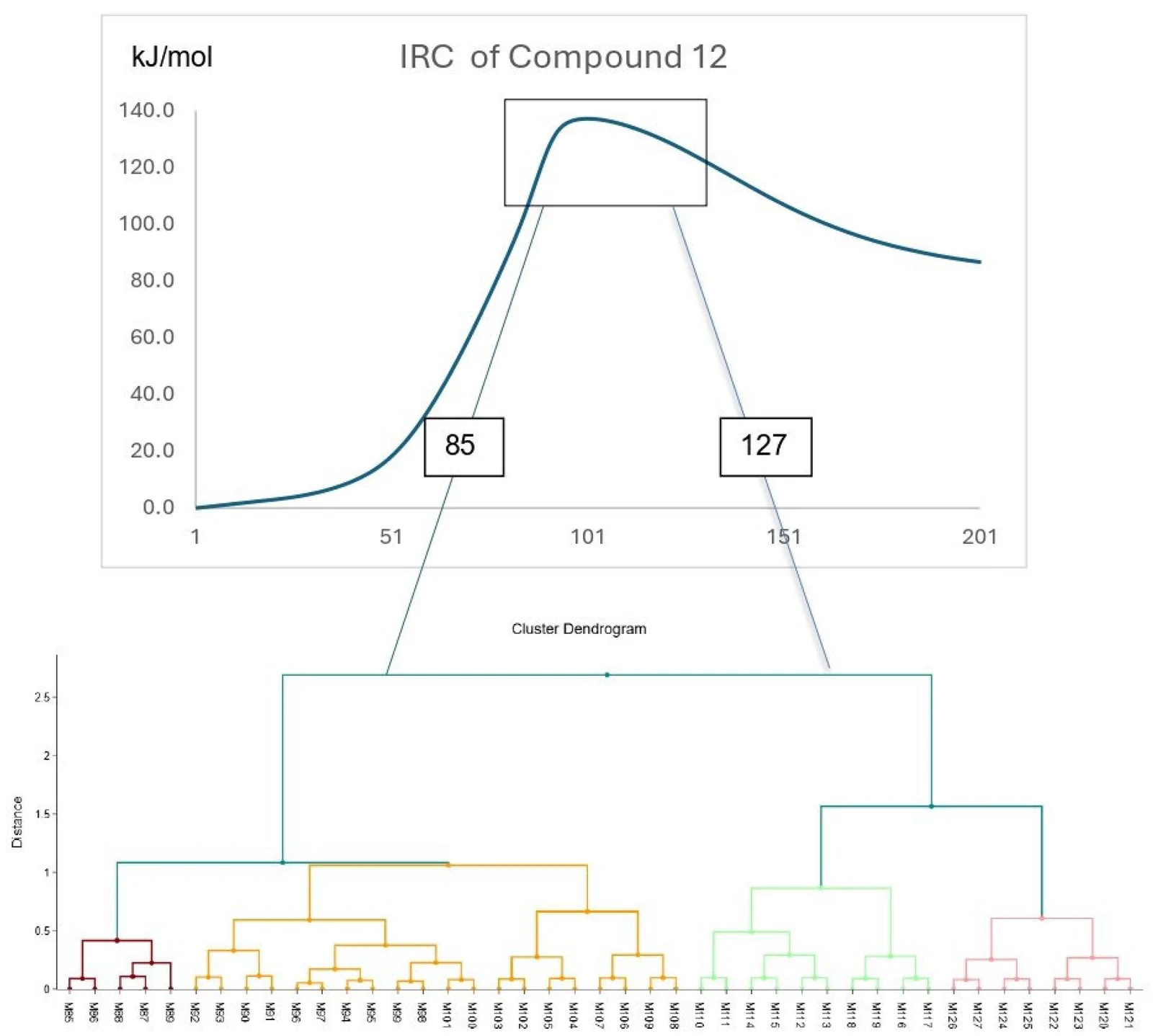
HCA dendrogram (HCA: complete-linkage, Euclidean: 4 clusters) for 61 points of the IRC of compound **12**, from M85 to M127 (30 points before and after the TS, M101), using 30 DFT variables (global descriptors, IBSI, electron densities and NPA charges) calculated at B97D-GD3BJ/Def2TZVP level of theory at 450 K. HCA dendrogram groupings show three blocks: reactants (M85–M100), TS (M101) and products (M102–M127).

The hierarchical cluster analysis method was applied to 30 DFT descriptors, including frontier orbital HOMO–LUMO, global reactivity descriptors, IBSI and NPA charges for 61 IRC points between M85 and M127 (30 points) and 30 points after the TS, M101. This selection focuses the analysis on the IRC regions with significant structural changes, i.e., proton transfer (associated with N4–H5 bond breaking and O2–H5 bond formation), and C3–C7 bond breaking. In contrast with energy diagrams like the IRC, HCA does not follow a chronological order of events. HCA groups points by chemical similarity, calculated as the Euclidian distance between vectors of 30 descriptors. Points with similar electronic profiles are fused at short distances (short fusion altitude); points with different electronic signatures are separated (high fusion altitude). A dendrogram is a diagram illustrating hierarchical clustering. The dendrogram created is a sensible diagnostic tool to detect how substituents reorganize the topology of the potential energy surface. Comparative analysis of three representative compounds, compound **12** (acetoacetamide), compound **1** (N-phenyl-3-oxobutanamide or acetoacetanilide), and compound **13** (N-phenyl-3-oxo-3-phenylpropanamide or 2-benzoylacetanilide), reveals a systematic trend that correlates the number of aromatic rings with the redistribution of points along the IRC, Figure 8 and Figure S5 for compounds **1** and **13**. The dendrogram of compound **12** (acetoacetamide), Figure 8, shows that the points are organized in three groups. Reactant points, M85–M100 for a compact cluster, fuse at shorter distances, compared to the rest of the dendrogram. In this region of high chemical similarity, IBSI bond indexes are close to the reactant values (IBSI_N4–H5 ≈ 0.72, IBSI_C3–C7 ≈ 1.02), and NPA charges are stable (O2 ≈ −0.54, N4 ≈ −0.78). The TS occupies a separate branch from the reactant block, joining with the rest of the tree at a fusion altitude higher than the height observed for the reactant points. This difference is due to the 30 descriptors reaching peak values at the saddle point: IBSI_O2–H5 = 0.91 (maximum), IBSI_N4–H5 = 0.32 and IBSI_C3–C7 = 0.45 (minimum), ΔEg gap HOMO–LUMO = 3.61 eV (global minimum), hardness η = 1.81 eV (minimum) and electrophilicity ω = 4.74 eV (maximum).

The electronic signature of the TS is clearly differentiated from the reactant region. The products region, M102–M127, is not a unique block; instead, there are different fusion heights, showing more variability than the reactant region. The dispersion observed for acetoacetamide, compound **12**, is a consequence of instability due to lack of resonance stabilization in the absence of phenyl groups, e.g., the NPA charges of N4 and O2 vary from (−0.87 to −0.89 and −0.62 to −0.64, respectively.

In compound **1** (acetoacetanilide), the presence of a phenyl substituent at the nitrogen substantially modifies the dendrogram topology (Figure S5). The TS shifts to a more centered position in the dendrogram, fusing earlier with the product and at intermediate fusion altitude, compared to compound **12**. This shift is due to stabilization by resonance of the negative charge at N4, developed during the proton transfer. In the TS region for compound **1**, charge values are less extreme, IBSI_O2–H5 lowers to 0.89, IBSI_N4–H5 increases to 0.35, IBSI_C3–C7 increases to 0.48, and qN4 becomes more negative (−0.88). The TS is chemically less extreme; the vector of descriptors is closer to products. The product region M102-M127 is more compact than compound **12**: fusion distances are shorter; however, the effect is less pronounced than compound **13**. This behavior reflects the effect of the phenyl group, stabilizing N4 charge, reduction conformational fluctuations and homogenizing descriptor values in the products.

Compound **13** (2-benzoylacetanilide), with two phenyl groups, has greater reorganization of the dendrogram. This shift is the result of the cumulative effect of the phenyl ring on N4 stabilizing the developing negative charge during the proton transfer, and the phenyl ring on C3 stabilizes the polarization of the alpha carbon, during the C3–C7 bond breaking. The TS descriptor values in compound **13** are less extreme compared to compounds **12** and **1**. The TS values are IBSI_O2–H5 = 0.87, IBSI_N4–H5 = 0.38, and IBSI_C3–C7 = 0.50, and the NPA charges are qN4 = −0.90 and q C3 = +0.71 (less positive than the TS charge on C3 for compounds **12** and **1**). The TS is chemically similar to the products; HCA places the TS and product on the same tree branch. The products region M102–M127, for a very compact cluster, had the lowest fusion distances in this study, indicating that the values of the product descriptors are very close. The resonance stabilization by two phenyl groups produces stable descriptor values; for example, the NPA charge, qN4, only varies from −0.90 to −0.91; qC3 between +0.70 and +0.71, IBSI_O2–H5 between 0.98 and 0.99, and IBSI_C3–C7 between 0.01 and 0.02. This homogeneity is seen in the close, compact HCA grouping.

HCA analysis utilizing 30 descriptors gives information about small structural changes along the reaction, which cannot be observed with Ea analysis alone. Parameters that influence the regrouping of points are bond indexes, IBSI (O2–H5, N4–H5 and C3–C7), and NPA charges, qN4, qO2, and qC3; these parameters show important changes along the IRC and are affected by the presence of phenyl groups.

**Figure 9:**
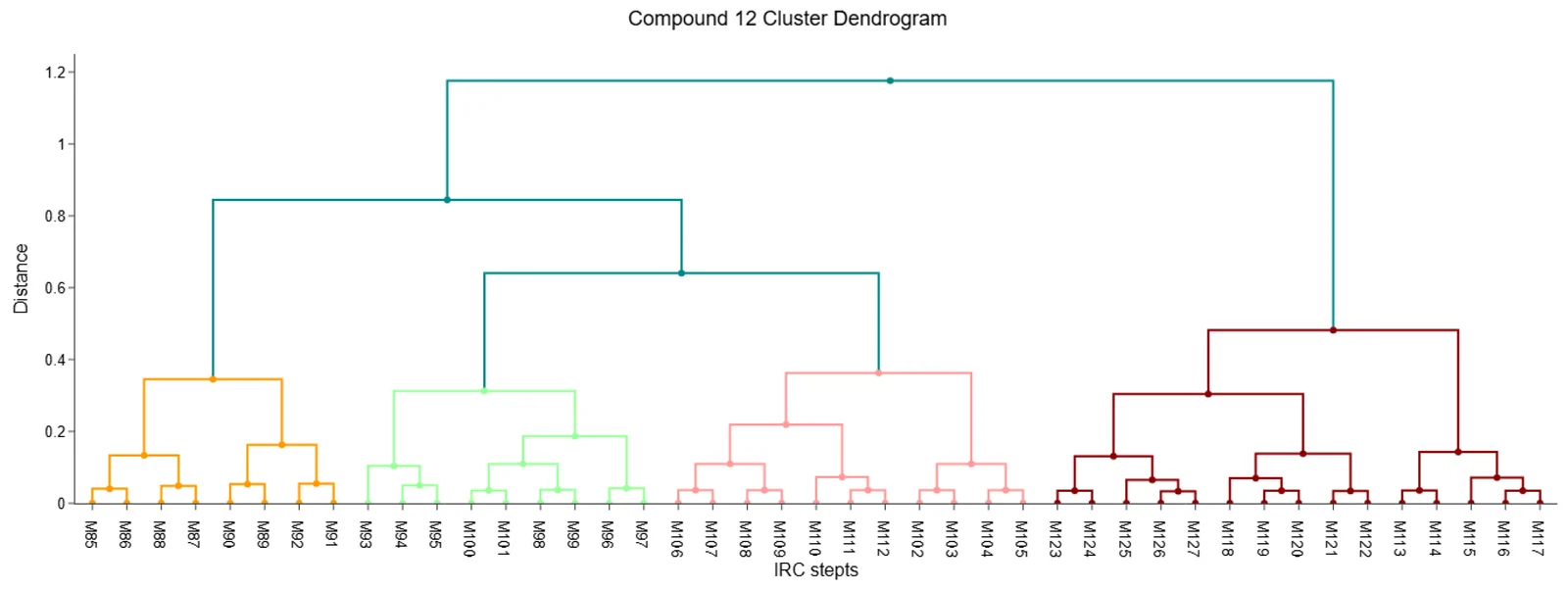
HCA dendrogram (HCA: complete-linkage, Euclidean: 4 clusters) for 61 IRC points for compound **12**, from (M85–M127), using only 13 variables with positive coefficients in PC1: (HOMO, LUMO, ΔEg gap, η, μ, IBSI_O2–H5, IBSI_N4–C3, IBSI_C7–C1, ρ_O2–H5, ρ_N4–C3, ρ_C7–C1, and NPA charges qC7, qC3, and qH5), calculated at B97D-GD3BJ/Def2TZVP level of theory at 450 K.

The HCA dendrogram produced for compound **12** using only 13 variables with positive coefficients in PC1 (Figure 9, Table 4) has essentially the same structure as the dendrogram generated with 30 variables (Figure 8). Reagents (M85–M100) form a compact cluster, the TS M101 is clearly separated, and the products (M102–127) have a dispersed distribution. This result implies that the variables selected from PC1 are determinant in the process: the capacity to polarize the electron cloud (associated with HOMO energy, LUMO energy, and ΔEg gap), the ability to transfer charge (associated with η and μ), the bond strength of bonds that break and form during the reaction (IBSI), and the charge redistribution (NPA) of the atoms involved in the TS. PCA not only reduces the dimensionality of the data but also identifies key parameters influencing the pyrolysis of acetoacetamide (compound **12**), thereby validating this method as a tool for investigating reaction mechanisms.

It is important to mention that our HCA analysis was performed on 61 IRC points (M85–M127) to focus on the region of significant structural changes. This window includes: (a) the final approach to the TS (M85–M100), (b) the TS itself (M101), and (c) the immediate product region (M102–M127). Analysis of all 201 points would unnecessarily emphasize the reactant region (M1–M84), where descriptor values change slowly, and the late product region (M128–M201), where values are already stabilized. The selected 61 points capture the chemically most interesting region while maintaining computational efficiency and clarity of visualization.

### 2.6. Dynamic Synchronicity, SyD

Traditionally, the degree of concertation in a reaction has been evaluated using Moyano’s synchronicity index [54], which is calculated from the changes in Wiberg bond indices between the TS (TS) and the reactants. Although this approach has been widely used, it offers a static and punctual view of a process that is, by nature, dynamic and evolutionary. To overcome this limitation and obtain a more complete characterization of the mechanism, in this work we introduce a new descriptor: Dynamic Synchronicity (*SyD*). This descriptor is calculated by applying the same Moyano equation (Equation (3)) to each of the 201 points along the intrinsic reaction coordinate (IRC), using the Wiberg indices obtained from NBO analysis.

This evolutionary approach allows us to build a complete synchronicity profile that reveals how the mechanism behaves at each stage of the process, from reactants to products. By analyzing these profiles for different compounds and mechanisms, we can extract mechanistic information that the static TS value cannot provide, making *SyD* a dynamic “fingerprint” of the reaction mechanism.

**Figure 10:**
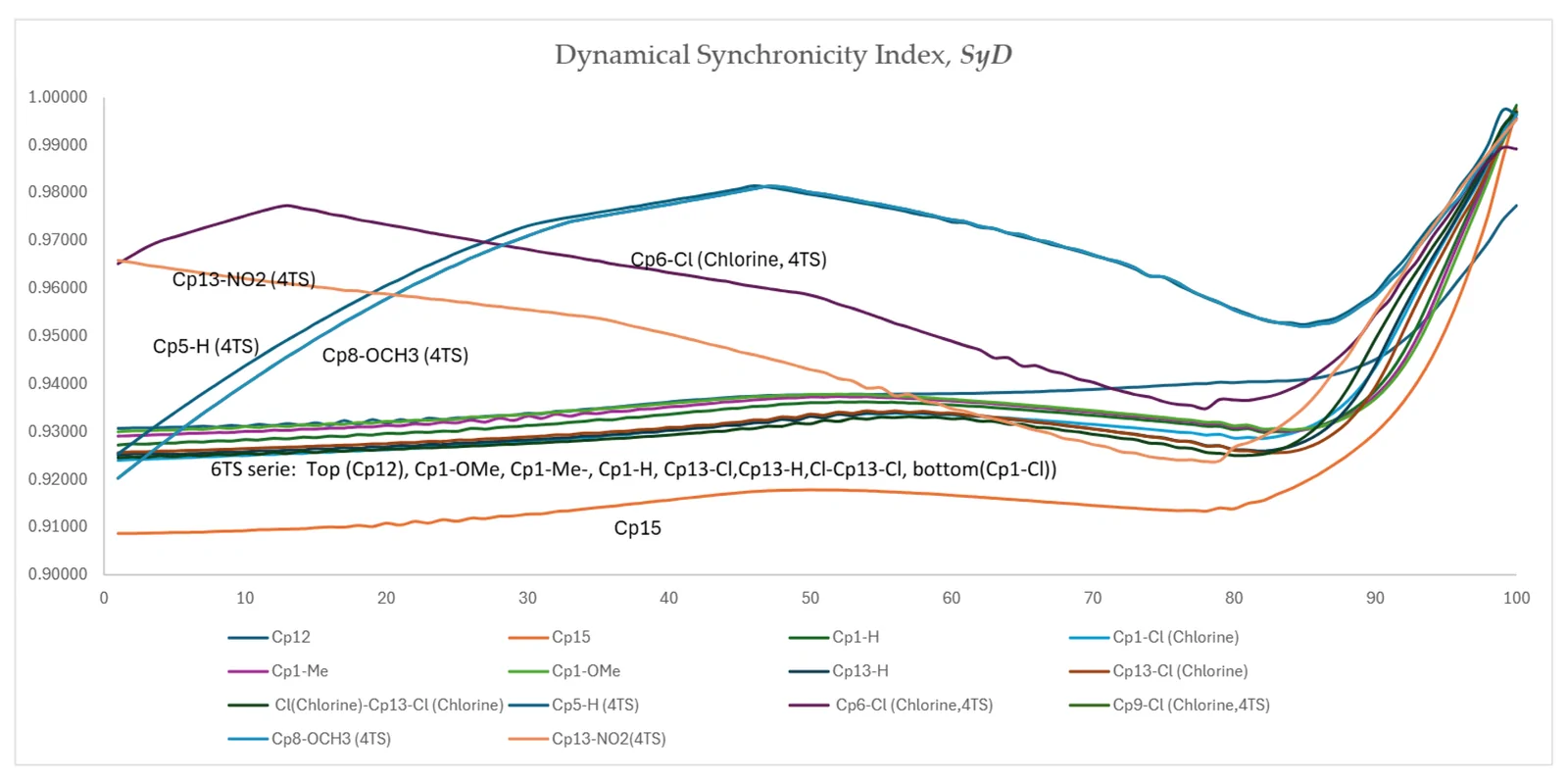
Evolution of the Dynamic Synchronicity (SyD) parameter, along the intrinsic reaction coordinate (IRC), for compounds in this work including reference compound **15**, derivatives containing EDGs (-Me, -Ome), derivatives with EWGs (-Cl, -NO2), variants 13a and 13b, and the four-member TS mechanism (4TS), calculated at B97D-GD3BJ/Def2TZVP level of theory at 450 K.

Figure 10 shows the *SyD* profiles for all compounds studied through the six-membered TS mechanism (6TS), while Table 5A,B present representative values of these profiles. A key finding is that all curves for the 6TS mechanism converge to the same global shape, characterized by a progressive increase in *SyD* from the reactant region (values ~0.93) to a maximum near the TS (values close to 0.99, point 100, Table 5A), followed by a slight decrease toward the products. This convergence indicates that, regardless of the substituent or the nature of the compound, the essence of the concerted six-membered mechanism remains the same. However, the exact position of the curve and its slope in the region near the TS are extremely sensitive to the electronic nature of the substituents. We observe that for compounds with electron-donating groups (EDGs, such as -Me and -OMe), the *SyD* profile shows a smoother and more sustained increase along the IRC, reaching higher values in the TS region (for example, *SyD* ≈ 0.998 for Cp1-OMe and SyD ≈ 0.996 for Cp1-Me at point 100, Table 5A). This behavior is the quantitative manifestation of a highly synchronous mechanism, where proton transfer (N4–H5 bond breaking and O2–H5 bond formation) and C3–C7 bond breaking occur almost simultaneously.

In contrast, for compounds with electron-withdrawing groups (EWGs, such as -Cl), the *SyD* profile is more abrupt and reaches notably lower values at the TS (for example, SyD ≈ 0.997 for Cp1-Cl at point 100, Table 5A). Although this decrease seems subtle, it is highly significant from a mechanistic point of view. It indicates that the mechanism becomes more asynchronous. EWGs destabilize the TS by failing to compensate for the developing partial charge, forcing the system to proceed more sequentially: proton transfer must be more advanced before C-C bond breaking can occur effectively. This behavior is even more pronounced in compound Cp13-NO_2_, where the curve clearly separates from the rest of the family, showing the destabilizing effect of the nitro group and confirming that the *SyD* descriptor can detect even subtle differences in mechanistic behavior.

The true power of the SyD descriptor becomes evident when comparing the 6TS mechanism with the alternative four-membered mechanism (4TS). In Figure 10, the *SyD* profiles for the 4TS mechanism (dashed lines) are not only significantly lower in magnitude in the TS region (values ~0.99, Table 5B), but they also do not converge to the same pattern. The shape of the curve varies drastically depending on the compound; only compounds Cp5-H and Cp8-OCH3 show a similar trajectory, while the rest show profiles with plateaus, inflexions, and erratic behaviors. This behavior is a direct reflection of the greater strain and conformational restriction of the four-membered ring. In this TS, the transfer of the amide proton to the α-carbon is a highly unfavorable event from an enthalpic point of view and is spatially demanding, requiring a rotation of approximately 90° of the hydrazone fragments (see Figure 3) to orient the α-carbon favorably. *SyD* captures this complexity. For example, compound Cp6-Cl (4TS) shows a profile with a pronounced plateau and values below 0.93 near the TS (Table 5B), indicating that bond breaking and formation are highly decoupled. The effect of the substituent is chaotic because the restricted geometry of the four-membered ring amplifies local steric and electronic interactions in a way that does not follow a simple linear trend, unlike what occurs with the 6TS mechanism.

**Figure 11:**
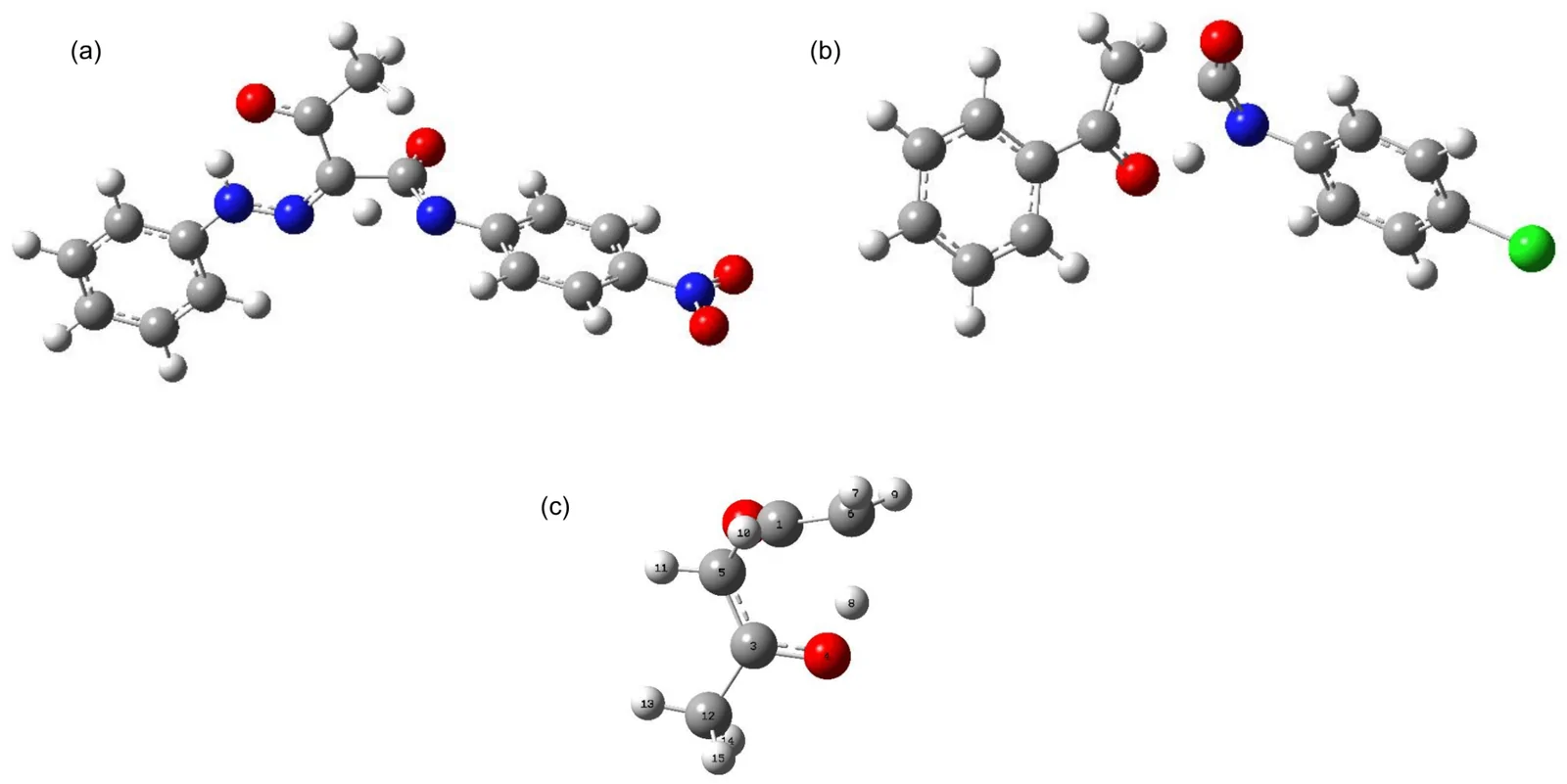
Structures of additional compounds: (**a**) Cp13-NO2-4TS, (**b**) Cp13-Cl (chlorine), and (**c**) the unsubstituted pentane-2,4-dione (cp15) incorporated into the SyD analysis in Figure 10.

To further validate the sensitivity and general applicability of the *SyD* descriptor, we extended the analysis to include additional compounds that were not part of the main thermodynamic study (Figure 11). These structures, Cp13-NO_2_ (4TS), Cp13-Cl (chlorine), and the unsubstituted pentane-2,4-dione (Cp15), were selected to test the descriptor’s response under extreme electronic conditions and in the absence of aromatic stabilization. The inclusion of these compounds serves multiple purposes. First, Cp15 (pentane-2,4-dione) represents the simplest structural scaffold, lacking the amide nitrogen and the aromatic rings present in the other compounds. Its *SyD* profile (Figure 10, Table 5A) shows a smooth and highly synchronous behavior, with values reaching 0.997 at the TS (point 100). This confirms that the fundamental six-membered proton transfer mechanism is inherently synchronous, and that the presence of the aryl groups in the other compounds modulates but does not alter this basic behavior. Second, Cp13-Cl (chlorine) was included to evaluate the effect of a chlorine substituent at a different position than in the main series. Interestingly, its *SyD* profile (Figure 10) closely follows the pattern of the other chlorinated compounds (Cp1-Cl and Cp6-Cl), demonstrating that the descriptor is sensitive to the electronic nature of the substituent (electron-withdrawing) rather than its specific position in the molecule. This positional independence reinforces the robustness of *SyD* as an electronic descriptor.

Third, and most importantly, Cp13-NO_2_ (4TS) was included to explore the behavior of *SyD* under an extreme electron-withdrawing condition within the four-membered mechanism. The resulting profile (Figure 10, Table 5B) shows the most asynchronous behavior of the entire series, with values dropping below 0.99 in the TS region and a markedly different shape compared to the other 4TS compounds. This indicates that the strong electron-withdrawing nature of the nitro group severely destabilizes the already constrained four-membered TS, forcing an even more sequential bond breaking/formation process. This result is consistent with the general trend observed for EWG substituents (more asynchronous mechanisms) and provides additional evidence that *SyD* can detect and quantify even extreme electronic perturbations.

The results presented validate *SyD* as a sensitive descriptor for the study of reaction mechanisms. They not only confirm that the 6TS mechanism is the predominant one (by showing more synchronous and energetically favorable profiles, Table 2) but also allow the classification and quantification of substituent effects in a way that was not possible with static indices. The ability of SyD to clearly differentiate between mechanisms (4TS vs. 6TS) and between families of substituent (EDG vs. EWG) positions makes it a powerful and versatile tool for mechanistic characterization in computational chemistry.

The implementation of *SyD* in this work provides an explanation of the pyrolysis mechanism of β-ketoamides and hydrazones and could be considered as possible groundwork for the study of a wide variety of concerted reactions, making it a significant methodological contribution to the field of computational chemistry.

The descriptor SyD introduced in this work represents a methodological contribution to the study of concerted reaction mechanisms. Its evolutionary approach along the reaction coordinate provides information complementary to traditional synchronicity indices. While its general applicability requires validation in other reaction systems, this approach may be particularly valuable in studying reactions with multiple reaction centers or processes where the degree of synchronicity varies significantly along the reaction coordinate. Further studies are needed to establish the general applicability of SyD to other classes of concerted reactions.

## 3. Computational Level of Theory

Initially, we used HCTH407 [55] for the series HCTH147, HCTH93, and HCTH [56], B97D3 [57], B97D [58], X3LYP [59], B3LYP [60], and CAM-B3LYP [61], with several basis sets to determine the best combination consistent with experimental activation energies. These methods are listed in Table S1, in the Supplementary Information.

We selected B97D3 and B97D functionals. B97D is a dispersion-corrected semi-local density functional (GGA) used in computational chemistry developed by Stefan Grimme to model large molecular systems, organic structures, and non-covalent van der Waals interactions. The functional B97D with the def2TZVP basis set [62] offered the best results combining precision and computational cost. For example, the calculated ΔH (~122–123 kJ/mol) and ΔG (~126 kJ/mol) are closer to the experimental average value (Ea 119.2 ± 17 kJ/mol). The basis set def2TZVP yields entropies ΔS ≈ −7 J/K mol, indicating that the functional/basis set B97D with def2TZVP is adequate to study the systems in this work, avoiding the cost of the use of larger basis sets. For example, def2TZVP affords comparable results with 6311G-++(2df,2pd), at a lower computational cost. We utilized SPARTAN26 and GAUSSIAN16 computational software [63,64].

## 4. Computational Methods

### 4.1. Calculation of Thermodynamic and Kinetic Parameters

Thermodynamic parameters were obtained from vibrational frequency calculations. Gibbs free energy values were calculated at 450 K and 1 atm, conditions used in the experimental work. Free energy of activation was obtained from the following:

(1) $$\Delta G^{‡}=G_{TS}-G_{Reactivos}$$

We calculated $\Delta H^{‡}$ and $\Delta S^{‡}$ in a similar fashion, using Equation (1). Rate constants for the gas-phase unimolecular decomposition were estimated using Eyring’s equation, assuming a transmission coefficient of κ=1. All calculations were performed at the reported experimental temperature, allowing direct comparison with kinetic data [65].

(2) $$k_{calc}(T)=\kappa \frac{k_{B}T}{h}exp(-\frac{\Delta G^{‡}}{RT})$$

We performed NBO analysis, Wiberg bond index, independent gradient model (IGM) for non-covalent interactions, and intrinsic reaction coordinate (IRC) calculations. The statistical multivariant model principal component analysis (PCA/HCA) was used to correlate quantum electronic descriptors with experimental values [66,67].

### 4.2. Natural Bond Orbital Analysis (NBO)

We carried out NBO calculations on the optimized geometries of the reactant, TS, and products [68]. From the NBO calculations, we obtained the atomic charges by natural population analysis (NPA). The redistribution of charges was analyzed along the reaction coordinate, focusing on the atoms involved in the proton transfer process, bond breaking and bond formation. Wiberg bond indexes ($W_{AB}$) were calculated to determine the effective bond order between atom pairs and evaluate the reaction progress in the TS.

The unimolecular pyrolysis reaction is concerted. To determine the synchronicity degree of this concerted process, we evaluated the evolution of Wiberg bond indexes along the intrinsic reaction coordinate (IRC). We specifically analyzed the bond indexes involved in bond breaking (C-N, N-H) and bond formation (O-H), following the changes from reactant to products.

The degree of concertation of the pyrolysis mechanism will be assessed by analyzing the evolution of the Wiberg binding indices along the intrinsic reaction coordinate (IRC). Bonds that are broken (C–N, N–H) and those that are formed (O–H) will be specifically analyzed, following their variation from reactants to products. The synchronicity index ($S_{y}$) is calculated as follows [54]:

(3) $$S_{y}=1-\frac{\sum_{i=1}^{n}|\delta B_{i}-{\delta B}_{v}|}{2(n-1){\delta B}_{v}}$$

where $\delta B_{i}$ is the change in bond index for bond *i*, between the reactant and TS, and ${\delta B}_{v}$ is the average change. An $S_{y}$ value close to 1 indicates a fully concerted, highly synchronous mechanism, whereas a value close to 0 indicates a fully asynchronous or stepwise process.

In this work, we determined the synchronicity value for each coupled pair among the reactant, TS, and product. We proposed a new descriptor, “Dynamic Synchronicity”, *SyD*, evaluating the synchronicity value for each compound along the intrinsic reaction coordinate (IRC) profile. We studied thirteen stable structures, totaling 201 points each. We studied the evolution of the synchronicity parameter along the reaction coordinate. The analysis of *Sy* along the IRC provides a dynamic perspective of the process, in contrast to the static view of conventional global descriptors. This novel approach to characterize the synchronicity of the mechanism at different stages of the process reveals characteristic patterns depending on the electronic nature of the substituents. This analysis is described in the Results section.

The Dynamic Synchronicity descriptor, SyD, is calculated by extending the rigorous mathematical foundation of the Moyano synchronicity index (Equation (3)) along the entire intrinsic reaction coordinate (IRC). While the traditional Moyano index provides a single value at the transition state, SyD evaluates synchronicity at multiple stages of the reaction, revealing how the mechanism evolves throughout the process.

### 4.3. Analysis of Non-Covalent Interactions

Topological analysis of the electron density (ED) provides local descriptors to characterize covalent and non-covalent interactions. The independent gradient model (IGM) method is used to identify, visualize, and quantify both covalent and non-covalent interactions within molecular systems by analyzing electron density gradients. It isolates intra-fragment and inter-fragment interactions to generate smooth 3D interaction isosurfaces. The descriptor Δg gives a local depiction of the electronic exchange and reveals clash regions. The method uses promolecular density. The promolecular density is a model of a molecule’s electron distribution formed by simply adding together the independent, spherically averaged charge densities of its constituent isolated atoms. It serves as a computationally fast reference state that excludes chemical bonding effects. Despite its limitations, the IGM method is a useful tool for qualitative and comparative analysis of non-covalent interactions in complex systems.

Electron density maps allow the visualization of regions with interactions in the TSs and compare the nature and intensity of the interaction between β-ketoamides and the arylhydrazone derivatives, providing evidence of stabilizing or destabilizing non-covalent interactions.

The intrinsic bond strength index (IBSI) and the independent gradient model (IGM) are used together to measure and visualize chemical bond strengths and molecular interactions. IBSI quantifies the amount of electronic exchange for unit length squared, normalized to 1 for the hydrogen molecule, H_2_ [69].

(4) $$\Delta g^{par}=\int_{V}\frac{\frac{{\delta g}^{par}}{d^{2}}dV}{\frac{{\delta g}^{\begin{array}{c} par\\ H_{2} \end{array}}}{d_{H_{2}}^{2}}dV}$$

### 4.4. Data Matrix Construction and Local and Global Reactivity Descriptors

We constructed a data matrix *X* of dimensions *n x p*, where n = 14, the number of compounds in this study, and p is a group of computational descriptors obtained from DFT calculations and analysis. Global descriptors depict molecular properties, not related to specific reactive sites in the molecule. Global descriptors are calculated from frontier orbital energies, ($E_{HOMO}$, $E_{LUMO}$). Structural, bond or local descriptors are represented by Wiberg bond indexes. NBO descriptors include atomic charge NPA.

Koopman’s theorem [67] approximates the HOMO and LUMO energies to the ionization potential I and electron affinity A, respectively, as follows:

(5) $$I=-E_{HOMO}\;y\;A=-E_{LUMO}$$

From *I* and *A* values, we can obtain the absolute electronegativity (χ), absolute hardness (η), chemical potential (μ), and softness (S, inverse of hardness):

(6) $$\chi =\frac{I+A}{2};\eta =\frac{I-A}{2};\mu =-\frac{I+A}{2};y\;S=\frac{1}{\eta }$$

Hardness (η), defined as η = (I − A)/2, is a fundamental CDFT descriptor that measures resistance to electron cloud polarization. In practice, η is approximated from frontier orbital energies as η ≈ (ELUMO − EHOMO)/2. For completeness, we report both η and the HOMO–LUMO gap ΔEg, as these descriptors capture related but subtly different aspects of electronic structure that may influence reactivity patterns.

Electrophilicity is a reactivity descriptor which quantifies the global electrophilicity of a molecule, on a relative scale. The Parr electrophilicity index (ω) is a quantitative measure of a chemical species’ power to accept electron density. It is defined as follows:

(7) $$\omega =\frac{\mu^{2}}{2\eta }$$

where μ is the electronic chemical potential, and η is the chemical hardness [70].

A good nucleophile is characterized by a low ionization potential I (corresponding to a high HOMO energy), which facilitates electron donation. A good electrophile is characterized by a low chemical potential μ (i.e., high electron affinity) and low hardness η, which facilitates electron acceptance. The electrophilicity index ω measures the tendency of a chemical species to accept electrons. This index assesses the energetic stabilization of a system when it acquires additional electron charge $∆N_{max}$ from its surroundings [71]; $∆N_{max}$ is calculated as follows:

(8) $$∆N_{max}=-\frac{\mu }{\eta }$$

While the electrophilicity index ω describes the tendency of a system to accept electrons, $∆N_{max}$ describes the capacity for additional electron charge of a given molecule.

Ayers et al. [72,73] proposed other reactivity descriptors, which measure the nucleophilic and electrophilic capacity of a leaving group, as nucleofugacity (Δ*E*ₙ), which ranks the ability of a leaving group to depart with a bonding pair of electrons, and electrofugacity (Δ*E*ₑ), defined as follows:

(9) $$∆E_{n}=-A+\omega \;y∆E_{e}=I+\omega$$

### 4.5. Principal Component Analysis (PCA) and Hierarchical Cluster Analysis (HCA)

Principal component analysis, PCA, and hierarchical cluster analysis, HCA, are the most widely used tools to explore similarities and hidden patterns among samples. PCA shrinks data size by transforming a pool of many variables into fewer, new combined variables. HCA sorts variables into groups shown as a tree diagram.

In PCA, the new, smaller group of variables constitutes the principal components. These components are obtained through orthogonal transformation. The principal components are orthogonal vectors of the covariance matrix. A covariance matrix is a square, symmetric table that shows the variance of separate variables on its diagonal and the covariance between different pairs of variables in its other spaces. It helps us see how items in a dataset change together. PCA is sensible to the scales of the original variables. For this reason, it is necessary to apply auto-scaling treatment. In this work, we used Numiqo for statistical calculations. Numiqo is a web app for statistical data analysis, available at https://www.numiqo.com [74].

To utilize these techniques, we need to calculate the standardized matrix Z, and from this, calculate the covariance matrix:

(10) $$C=\frac{1}{n-1}Z^{T}Z$$

This will provide the autovectors (*V*), which define the directions of the principal components, and the auto values ($\lambda_{j}$), which quantify the variance for each component.

The scores (T = ZV) are analyzed to identify possible grouping in β-ketoamides and arylhydrazone derivatives, while the loadings allow the determination of which descriptors contribute predominantly to the observed differences in reactivity [75,76,77].

Hierarchical cluster analysis (HCA) represents the available data in 2D dendrograms. A dendrogram is a tree-like diagram that visualizes hierarchical clustering, showing how data points or groups are merged or split during hierarchical cluster analysis. Its relationship with a similarity matrix forms the mathematical foundation for identifying natural groupings in data. It displays individual items at the base and progressive group connections moving upward. To build a dendrogram, the distances between pairs are calculated, and the similarity matrix is created. For two samples k and l, the similarity index is calculated as follows:

(11) $$S_{kl}=1-\frac{d_{kl}}{d_{max}}$$

where $d_{kl}$ is the Euclidean distance between samples k and l, and $d_{max}$ is the maximum distance between any two samples in the dataset.

## 5. Conclusions

In this work, we carried out an exhaustive computational study of the gas-phase pyrolysis of N-aryl-3-oxobutanamides (β-ketoamides) and 2-arylhydrazone derivatives, employing density functional theory at the B97D-GD3BJ/Def2TZVP level, multivariant analysis PCA and HCA. Our computational results are consistent with the proposed concerted asynchronous mechanism through a six-membered cyclic transition state. The IRC calculations show a single, continuous reaction pathway without detectable intermediates, supporting the classification as a single-step process. The Wiberg bond index evolution demonstrates that bond breaking and formation events occur sequentially within the same elementary step, consistent with an asynchronous concerted mechanism. Our Wiberg bond index analysis along the IRC reveals that the N-H bond rupture (and concomitant O-H bond formation) is already significantly advanced at the TS, while the C-C bond cleavage is less advanced. This observation indicates that the activation energy barrier is primarily associated with the C-C bond breaking event, which represents the rate-determining step within the concerted asynchronous mechanism. The N-H bond rupture occurs earlier along the reaction coordinate, as reflected in the lower IBSI values for N-H at the TS compared to the reactants. We introduced the concept of Dynamic Synchronicity, *SyD*, as a new descriptor to differentiate substituent effects in concerted mechanisms. In the gas-phase pyrolysis of β-ketoamides and arylhydrazone derivatives, we observed that electron-donor substituents (e.g., Me and OMe) tend to facilitate more synchronous mechanisms, whereas electron-withdrawing substituents (e.g., Cl and NO_2_) tend to favor more asynchronous processes and destabilize the TS. These trends are consistent with the observed differences in reactivity and with the calculated and experimental activation energies for the set of compounds studied. Principal component analysis (PCA) revealed that PC1, which groups variables such as HOMO, LUMO energies, hardness, chemical potential and bond indexes, is the determinant for reactivity. PCA can be used to identify patterns for reactivity-stability using a training set of molecules with available experimental data. This information can then be used to evaluate the reactivity in other molecules outside the training set. The strategy presented in this work can be used to predict reactivity/stability of a variety of systems. Hierarchical cluster analysis (HCA) allowed the visualization of how the organization of the IRC points is modified by the substituents. Subtle effects in the reaction’s dynamics can be analyzed. This work provides a solid theoretical validation of the reaction mechanism and proposes an integrated methodology combining computational tools with chemometric techniques, offering a more complete approach to studying chemical reactivity. The selection criteria of the variables derived from PCA and HCA can be useful in the rational design of compounds with the highest thermal stability. The proposed descriptor, Dynamic Synchronicity, *SyD*, provides a novel global-evolutive parameter to study the reaction mechanism.

## Figures and Tables

**Table 1:** Experimental Arrhenius parameters and energies of activation at 450 K [23].

Compound		Log A (s^−1^)	E_a_ (kJ mol^−1^)	k (10^3^ s^−1^)
Ketoanilides				
**1**	N-phenyl	10.22 ± 0.39	106.1 ± 2.93	8.13
**2**	p-Cl-phenyl	14.0 ± 1.4	136.1 ± 1.35	16.2
**3**	p-Me-phenyl	9.97 ± 0.01	105.0 ± 0.012	6.02
**4**	p-OMe-phenyl	10.39 ± 0.24	110.4 ± 1.92	3.80
**12**	Acetoacetamide	11.76 ± 0.05	124.3 ± 3.04	2.14
**13**	N-phenyl-**3**-oxo-**3**-phenylpropanamide	9.91 ± 0.56	102.2 ± 4.56	11.2
**14**	N,N’-diphenylpropanediamide	10.69 ± 0.19	131.7 ± 1.80	2.57
Arylhydrazone Derivatives				
**5**	N-phenyl-**2**-phenylhydrazone	12.97 ± 0.16	158.70 ± 1.63	0.0035
**6**	p-Cl-phenyl/**2**-phenylhydrazone	13.19 ± 0.57	161.30 ± 5.44	0.0029
**7**	p-Me-phenyl/**2**-phenylhydrazone	13.49 ± 0.82	164.70 ± 8.68	0.0024
**8**	p-OMe-phenyl/**2**-phenylhydrazone	13.28 ± 0.09	162.30 ± 0.92	0.0028
**9**	N-phenyl-**2**-p-Cl-phenylhydrazone	13.59 ± 0.34	162.70 ± 3.14	0.0051
**10**	N-phenyl-**2**-p-Me-phenylhydrazone	13.15 ± 0.36	159.60 ± 3.51	0.0043
**11**	N-phenyl-**2**-p-OMe-phenylhydrazone	12.30 ± 0.20	148.90 ± 2.22	0.0011

Arrhenius parameters for β-ketoamides are, on average, log A (s^−1^) = 12.0 ± 2.0 and E_a_ = 119.2 ± 17.0 kJ mol^−1^. Arrhenius parameters for arylhydrazone derivatives are log A (s^−1^) = 13.0 ± 0.7 and significatively larger energies of activation, E_a_ = 157.5 ± 8.6 kJ mol^−1^.

**Table 2:** Calculated thermodynamic parameters using B97D-GD3BJ/Def2TZVP level of theory, at 450 K. Substituent G information for each compound is available in Scheme 3; for example, for compound **1**, G=H.

Structure	Compound		ΔH^‡^ (kJ/mol)	ΔG^‡^ (kJ/mol)	ΔS^‡^ (J/Kmol)	Ea (kJ/mol)
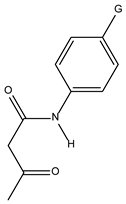	**1**	Six-member TS	122.93	126.14	−7.15	126.67
	**2**		123.86	126.87	−6.69	127.60
	**3**		122.76	126.90	−9.20	126.50
	**4**		122.72	126.60	−8.63	126.46
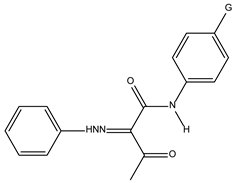	**5**	Six-member TS	174.54	161.90	28.11	178.28
	**6**		176.79	163.40	29.77	180.53
	**7**		174.15	161.53	28.06	177.89
	**8**		173.68	162.06	25.84	177.42
	**5**	Four-member TS	185.58	185.02	1.24	189.32
	**6**		184.28	183.23	2.33	188.02
	**7**		186.11	185.40	1.58	189.85
	**8**		185.44	183.43	4.47	189.18

**Table 3:** Thermodynamic parameters at B97D-GD3BJ/Def2TZVP level of theory, and 450 K.

Structure	Compound			ΔH^‡^ (kJ/mol)	ΔG^‡^ (kJ/mol)	ΔS^‡^ (J/Kmol)	Ea (kJ/mol)
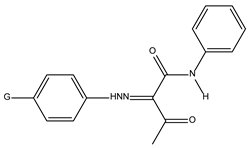	**9**	Six-member TS		175.55	150.15	56.49	179.29
	**10**			178.10	150.71	60.91	181.84
	**11**			177.68	163.46	31.60	181.42
	**9**		Four-member TS	187.89	174.66	29.43	191.64
	**10**			185.49	173.82	26.59	189.23
	**11**			179.17	179.28	−0.26	182.91
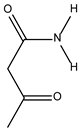	**12**		Six-member TS	126.13	126.72	−1.31	129.87
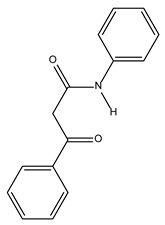	**13**		Six-member TS	120.98	112.40	19.08	124.72
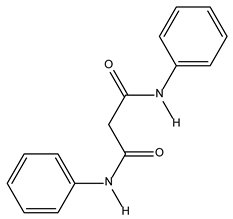	**14**		Six-member TS	148.98	214.69	−4.62	152.72
				146.52	203.40	15.04	150.27

**Table 4:** Principal component PC1 contributions for 201 IRC points of compounds Cp12, Cp13, Cp1-H, Cp1-Cl, Cp1-Me, and Cp13-Cl (chlorine). For simplicity, the abbreviation Cp is used hereafter to denote each compound, thereby avoiding any ambiguity with atomic indices or labels.

Variable	Compounds					
	Cp12	Cp13	Cp1-H	Cp1-Cl	Cp1-Me	Cp13-Cl
HOMO (eV)	0.92	0.37	−0.07	−0.47	−0.67	0.29
LUMO (eV)	0.94	0.95	0.94	0.93	0.95	0.95
Gap ΔEg (eV)	0.89	0.97	0.94	0.93	0.96	0.97
I	−0.92	−0.37	0.07	0.47	0.67	−0.29
A	−0.94	−0.95	−0.94	−0.93	−0.95	−0.95
X	−0.93	−0.91	−0.86	−0.85	−0.86	−0.90
η	0.89	0.97	0.94	0.93	0.96	0.97
μ	0.93	0.91	0.86	0.85	0.86	0.90
S	−0.88	−0.96	−0.94	−0.92	−0.96	−0.97
ω	−0.94	−0.96	−0.95	−0.93	−0.95	−0.97
ΔN	−0.94	−0.97	−0.95	−0.93	−0.96	−0.97
ΔEn	−0.95	−0.96	−0.94	−0.92	−0.95	−0.96
ΔEe	−0.94	−0.95	−0.93	−0.91	−0.93	0.98
IBSI_O2-H5	0.97	0.98	0.98	0.97	0.98	−0.97
IBSI_H5-N4	−0.95	−0.96	−0.96	−0.95	−0.96	0.99
IBSI_N4-C3	0.99	0.99	0.99	0.98	0.99	−0.97
IBSI_C3-C7	−0.95	−0.96	−0.96	−0.95	−0.96	0.99
IBSI_C7-C1	0.98	0.98	0.98	0.98	0.98	−0.98
IBSI_C1-O2	−0.98	−0.98	−0.98	−0.97	−0.98	0.98
ρ_O2-H5	0.97	0.98	0.98	0.97	0.98	−0.97
ρ_H5-N4	−0.95	−0.96	−0.96	−0.95	−0.96	0.99
ρ_N4-C3	0.98	0.98	0.98	0.98	0.98	−0.96
ρ_C3-C7	−0.94	−0.95	−0.95	−0.95	−0.95	0.98
ρ_C7-C1	0.96	0.97	0.97	0.96	0.97	−0.99
ρ_C1-O2	−0.98	−0.98	−0.99	−0.98	−0.98	−1.00
qC1	−1.00	−1.00	−1.00	−0.99	−1.00	0.99
qC7	0.97	1.00	0.99	0.98	0.98	0.98
qC3	0.97	0.98	0.98	0.98	0.99	−0.23
qN4	−0.32	−0.17	−0.28	−0.23	−0.32	0.97
qH5	0.94	0.96	0.96	0.96	0.97	−0.98
qO2	−0.98	−0.98	−0.99	−0.98	−0.99	0.29

**Table 5:** (A) Sample *SyD* values evaluated along the IRC, utilizing Wiberg bond indexes and NBO, calculated at GD3BJ/Def2TZVP level of theory at 450 K. (B) Sample *SyD* values, as defined in Table 5A. The notation 4TS refers to a reaction mechanism involving a four-membered ring TS.

**(A)**							
**IRC PAIR**	**Cp12**	**Cp15**	**Cp1-H**	**Cp1-Cl (Chlorine)**	**Cp1-Me**	**Cp1-OMe**	**Cp13-H**
1	0.93068	0.90866	0.92718	0.92397	0.92904	0.92997	0.92502
2	0.93076	0.90867	0.92741	0.92417	0.92918	0.93013	0.92532
3	0.93079	0.90874	0.92737	0.92420	0.92923	0.93016	0.92523
4	0.93083	0.90880	0.92760	0.92437	0.92937	0.93032	0.92542
5	0.93095	0.90887	0.92760	0.92444	0.92943	0.93031	0.92525
6	0.93094	0.90891	0.92787	0.92454	0.92961	0.93054	0.92538
7	0.93116	0.90896	0.92782	0.92472	0.92966	0.93046	0.92543
8	0.93096	0.90909	0.92808	0.92475	0.92981	0.93081	0.92565
9	0.93132	0.90917	0.92800	0.92497	0.92982	0.93067	0.92571
10	0.93109	0.90925	0.92834	0.92496	0.93007	0.93103	0.92578
51	0.93778	0.91777	0.93608	0.93318	0.93722	0.93773	0.93289
52	0.93777	0.91779	0.93620	0.93358	0.93737	0.93785	0.93364
53	0.93782	0.91768	0.93616	0.93352	0.93724	0.93772	0.93338
54	0.93781	0.91762	0.93624	0.93376	0.93733	0.93772	0.93392
55	0.93780	0.91747	0.93614	0.93366	0.93714	0.93753	0.93379
56	0.93784	0.91736	0.93613	0.93386	0.93711	0.93746	0.93404
57	0.93786	0.91719	0.93598	0.93367	0.93692	0.93731	0.93390
58	0.93788	0.91704	0.93589	0.93376	0.93682	0.93717	0.93394
59	0.93792	0.91687	0.93571	0.93353	0.93661	0.93694	0.93369
60	0.93794	0.91667	0.93559	0.93355	0.93645	0.93679	0.93365
61	0.93800	0.91648	0.93538	0.93331	0.93622	0.93653	0.93335
91	0.94685	0.93269	0.94213	0.94845	0.94064	0.93982	0.94978
92	0.94909	0.93627	0.94664	0.95401	0.94466	0.94360	0.95556
93	0.95170	0.94047	0.95228	0.95991	0.94992	0.94842	0.96101
94	0.95486	0.94547	0.95871	0.96536	0.95622	0.95440	0.96615
95	0.95835	0.95150	0.96516	0.97056	0.96333	0.96125	0.97100
96	0.96215	0.95852	0.97181	0.97569	0.97037	0.96840	0.97608
97	0.96580	0.96658	0.97816	0.98066	0.97719	0.97546	0.98119
98	0.96967	0.97552	0.98461	0.98590	0.98357	0.98272	0.98677
99	0.97422	0.98685	0.99251	0.99242	0.99081	0.99050	0.99375
100	0.97734	0.99757	0.99833	0.99685	0.99565	0.99656	0.99692
**(B)**							
**IRC PAIR**	**Cl (Chlorine)-Cp13-Cl (Chlorine)**		**Cp5-H (4TS)**	**Cp6-Cl (Chlorine, 4TS)**	**Cp9-Cl (Chlorine, 4TS)**	**Cp8-OCH3 (4TS)**	**Cp13-NO2 (4TS)**
1	0.92453		0.92534	0.9652	0.9203	0.9203	0.9658
2	0.92465		0.92755	0.9669	0.9226	0.9226	0.9654
3	0.92461		0.92975	0.9686	0.9249	0.9249	0.9649
4	0.92492		0.93188	0.9699	0.9272	0.9272	0.9645
5	0.92477		0.93395	0.9708	0.9294	0.9294	0.9640
6	0.92507		0.93598	0.9717	0.9316	0.9316	0.9636
7	0.92495		0.93800	0.9726	0.9338	0.9338	0.9632
8	0.92527		0.93993	0.9734	0.9358	0.9358	0.9628
9	0.92514		0.94187	0.9743	0.9379	0.9379	0.9624
10	0.92547		0.94378	0.9752	0.9399	0.9399	0.9620
51	0.93236		0.97924	0.9578	0.9797	0.9797	0.9424
52	0.93214		0.97878	0.9567	0.9792	0.9792	0.9411
53	0.93279		0.97824	0.9558	0.9786	0.9786	0.9406
54	0.93263		0.97771	0.9548	0.9780	0.9780	0.9391
55	0.93303		0.97710	0.9538	0.9776	0.9776	0.9392
56	0.93292		0.97661	0.9527	0.9770	0.9770	0.9375
57	0.93312		0.97594	0.9518	0.9764	0.9764	0.9378
58	0.93295		0.97533	0.9508	0.9757	0.9757	0.9364
59	0.93300		0.97482	0.9499	0.9750	0.9750	0.9359
60	0.93266		0.97390	0.9490	0.9744	0.9744	0.9348
61	0.93273		0.97373	0.9480	0.9740	0.9740	0.9343
91	0.95447		0.96259	0.9575	0.9614	0.9614	0.9592
92	0.95942		0.96582	0.9622	0.9643	0.9643	0.9633
93	0.96383		0.96968	0.9659	0.9685	0.9685	0.9684
94	0.96836		0.97348	0.9706	0.9722	0.9722	0.9726
95	0.97261		0.97680	0.9745	0.9760	0.9760	0.9768
96	0.97738		0.98146	0.9788	0.9792	0.9792	0.9808
97	0.98235		0.98527	0.9831	0.9838	0.9838	0.9847
98	0.98774		0.99018	0.9865	0.9879	0.9879	0.9883
99	0.99370		0.99723	0.9895	0.9913	0.9913	0.9919
100	0.99691		0.99634	0.9892	0.9963	0.9963	0.9954

## Data Availability

All computational details and data are available in a Supplementary File.

## Supplementary Materials

The following supporting information can be downloaded at: https://www.mdpi.com/article/10.3390/molecules31183201/s1.
