# Supplementary material for: Study of the Gas-Phase Pyrolysis of N-Aryl-3-Oxobutanamides and the 2-Aryl Hydrazone Derivatives: A Novel Computational Approach Including DFT with Multivariant Analysis
Source: Molecules. 2026 Sep 10;31(18):3201. doi: 10.3390/molecules31183201 (PMC13609908; doi:10.3390/molecules31183201)
Supplement: Supplementary file 1 [file molecules-31-03201-s001.zip › molecules-4525522-supplementary.pdf]

# Supplementary MATERIAL

## STUDY OF THE GAS-PHASE PYROLYSIS OF N-ARYL-3-OXOBUTANAMIDES AND THE 2-ARYL HYDRAZONE DERIVATIVES: A NOVEL COMPUTATIONAL APPROACH INCLUDING DFT WITH MULTIVARIANT ANALYSIS

Rosalinda Ipanaque-Chávez [1], Marcos Loroño [1,] *, Tania Cordova-Sintjago [2], José L. Paz [3] y Cecilio Julio Alberto Garrido Schaeffer [4]. *Correspondence author: **mloronog@unmsm.edu.pe**

**Table S1.** Preliminary calculations on compound 1, G = H, Figure 5. Choice of model. Only HCTH series models did not include GD3BJ type dispersion correction.

| Functional | %HF | basis set | ΔH (kJ/mol) | ΔG (kJ/mol) | ΔS (J/Kmol) | Ea (kJ/mol) | Ea (kJ/mol) |
|---|---|---|---|---|---|---|---|
| HCTH407 | 0 | 631G-d | 151.68 | 152.46 | -1.74 | 155.42 | 116.1 ± 4 Experimental, Table 1. |
| HCTH407 | 0 | 631G-d,p | 144.72 | 145.18 | -1.04 | 148.46 | |
| HCTH407 | 0 | 631G-d,2p | 144.47 | 145.09 | -1.37 | 148.21 | |
| HCTH407 | 0 | 631G-d,3p | 137.20 | 138.15 | -2.10 | 140.94 | |
| HCTH407 | 0 | 631G-d,pd | 143.19 | 143.84 | -1.44 | 146.93 | |
| HCTH407 | 0 | 631G-d,2pd | 143.10 | 143.74 | -1.42 | 146.84 | |
| HCTH407 | 0 | 6311G-d,p | 141.59 | 142.83 | -2.76 | 145.33 | |
| HCTH407 | 0 | 6311G-++d,p | 142.23 | 145.52 | -7.30 | 145.97 | |
| HCTH407 | 0 | def2TZVP | 138.54 | 141.34 | -6.23 | 142.28 | |
| HCTH407 | 0 | def2TZVP | 138.54 | 141.34 | -6.23 | 142.28 | |
| HCTH407 | 0 | 6311G-++2d,2p | 141.66 | 145.51 | -8.56 | 145.40 | |
| HCTH147 | 0 | def2TZVP | 133.84 | 135.93 | -4.66 | 137.58 | |
| HCTH93 | 0 | def2TZVP | 135.83 | 138.27 | -5.43 | 139.57 | |
| HCTH | 0 | 6311G-++2df,2pd | 131.08 | 133.25 | -4.84 | 134.82 | |
| HCTH | 0 | 6311G-++2d,2p | 133.55 | 135.49 | -4.32 | 137.29 | |
| HCTH | 0 | def2TZVP | 130.28 | 131.74 | -3.24 | 134.02 | |
| B97D3 | 0 | def2TZVP | 120.83 | 126.15 | -7.16 | 124.57 | |
| B97D3 | 0 | def2TZVP | 122.93 | 126.14 | -7.14 | 126.67 | |
| B97D3 | 0 | 6311G-++2df,2pd | 123.29 | 127.41 | -9.15 | 127.03 | |
| B97D3 | 0 | 6311G-++3df,3pd | 123.24 | 127.29 | -9.01 | 126.98 | |
| B97D | 0 | 6311G-++2df,2pd | 123.29 | 127.42 | -9.19 | 127.03 | |
| B97D | 0 | def2TZVP | 122.93 | 126.14 | -7.15 | 126.67 | |
| B97D | 0 | 6311G-++3df,3pd | 136.13 | 140.55 | -9.83 | 139.87 | |

| | | | | | | | |
|---|---|---|---|---|---|---|---|
| X3LYP | 21.8 | def2TZVP | 154.74 | 157.47 | -6.08 | 158.48 | |
| B3LYP | 20 | def2TZVP | 149.45 | 152.43 | -6.64 | 153.20 | |
| B3LYP | 20 | 6311G-++2df,2pd | 149.19 | 152.99 | -8.45 | 152.93 | |
| CAM-B3LYP | 0-100 | def2TZVP | 169.86 | 172.72 | -6.36 | 173.60 | |
| WB97XD | 19-65 | 6311G-++2df,2pd | 173.15 | 176.66 | -7.80 | 176.89 | |
| WB97XD | 19-65 | def2TZVP | 173.47 | 176.19 | -6.05 | 177.21 | |
| B97D3 | 20 | 6311G-++3df,3pd | 282.74 | 268.43 | 31.82 | 286.48 | |
| B97D3 | 20 | def2TZVP | 282.13 | 267.51 | 32.51 | 285.87 | |
| B97D | 0 | def2TZVP | 282.13 | 267.53 | 32.47 | 285.87 | |
| B97D | 0 | 6311G-++3df,3pd | 282.72 | 268.48 | 31.66 | 286.46 | |

**Table S2.** Sample of energy calculations of HOMO and LUMO and breccia (ΔEg), in units of eV, calculated at DFT level B97D-GD3BJ/Def2TZVP in gas phase. With its global descriptors, compound 12: acetoacetamide.

| IRC Steps | Gap (eV) | HOMO (eV) | LUMO (eV) | I | A | X |
|---|---|---|---|---|---|---|
| M1 | 3.7514 | -5.8638 | -2.1124 | 5.8638 | 2.1124 | 3.9881 |
| M2 | 3.7546 | -5.8638 | -2.1092 | 5.8638 | 2.1092 | 3.9865 |
| M3 | 3.7397 | -5.8649 | -2.1252 | 5.8649 | 2.1252 | 3.9951 |
| … | … | … | … | … | … | … |
| M70 | 3.7914 | -6.0094 | -2.2180 | 6.0094 | 2.2180 | 4.1137 |
| M71 | 3.7944 | -6.0056 | -2.2112 | 6.0056 | 2.2112 | 4.1084 |
| … | … | … | … | … | … | … |
| M101 | 3.6148 | -5.9471 | -2.3323 | 5.9471 | 2.3323 | 4.1397 |
| M102 | 3.6278 | -5.9424 | -2.3146 | 5.9424 | 2.3146 | 4.1285 |
| M103 | 3.6409 | -5.9364 | -2.2956 | 5.9364 | 2.2956 | 4.1160 |
| … | … | … | … | … | … | … |
| M201 | 3.9497 | -5.1947 | -1.2449 | 5.1947 | 1.2449 | 3.2198 |

| IRC Steps | η | μ | S | ω | ΔN | ΔEn | ΔEe |
|---|---|---|---|---|---|---|---|
| M1 | 1.8757 | -3.9881 | 0.5331 | 4.2397 | 2.1262 | 2.1273 | 10.1035 |
| M2 | 1.8773 | -3.9865 | 0.5327 | 4.2327 | 2.1235 | 2.1235 | 10.0965 |
| M3 | 1.8699 | -3.9951 | 0.5348 | 4.2678 | 2.1366 | 2.1426 | 10.1327 |
| … | … | … | … | … | … | … | …. |
| M70 | 1.8957 | -4.1137 | 0.5275 | 4.4634 | 2.1700 | 2.2454 | 10.4728 |
| M71 | 1.8972 | -4.1084 | 0.5271 | 4.4484 | 2.1655 | 2.2372 | 10.4540 |
| … | … | … | … | … | … | … | …. |
| M101 | 1.8074 | -4.1397 | 0.5533 | 4.7408 | 2.2904 | 2.4085 | 10.6879 |
| M102 | 1.8139 | -4.1285 | 0.5513 | 4.6983 | 2.2760 | 2.3837 | 10.6407 |
| M103 | 1.8204 | -4.1160 | 0.5493 | 4.6532 | 2.2610 | 2.3576 | 10.5896 |

| | | | | | | | |
|---|---|---|---|---|---|---|---|
| ... | ... | ... | ... | ... | ... | ... | .... |
| M201 | 1.9749 | -3.2198 | 0.5064 | 2.6247 | 1.6304 | 1.3798 | 7.8194 |

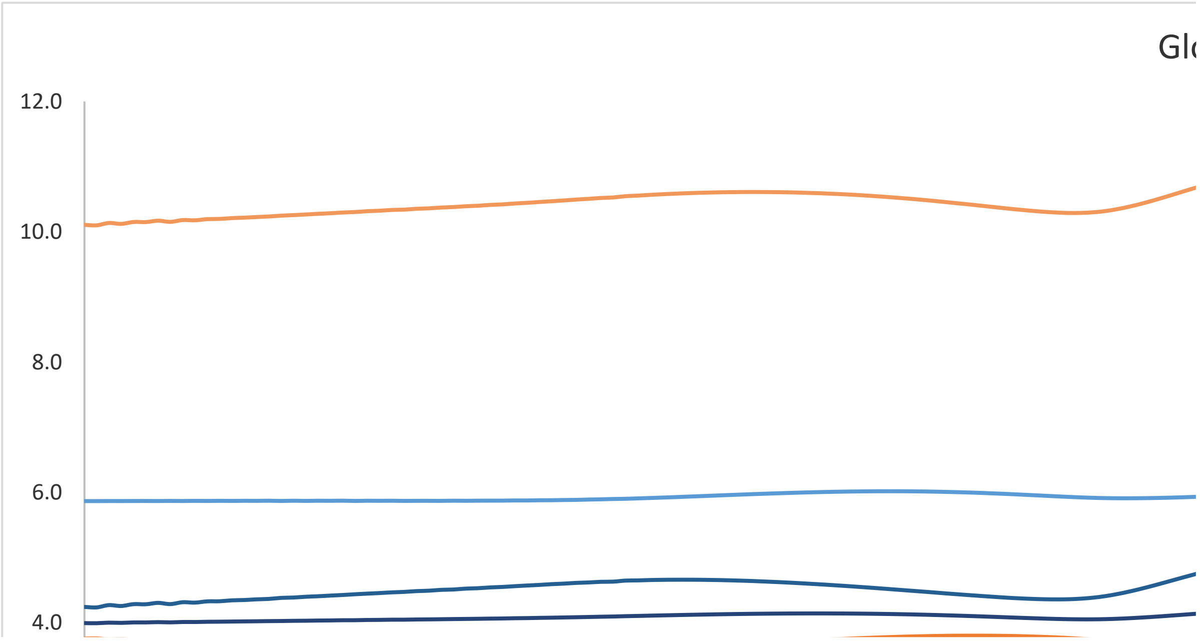


**Figure S1.** Calculations of the global descriptors that depend on the energies of the boundary orbitals Homo and Lumo, corresponding to compound 12, GD3BJ/Def2TZVP at 450 K. The values are in eV units; the x-axis corresponds to the reaction coordinate of the IRC analysis (201 points).

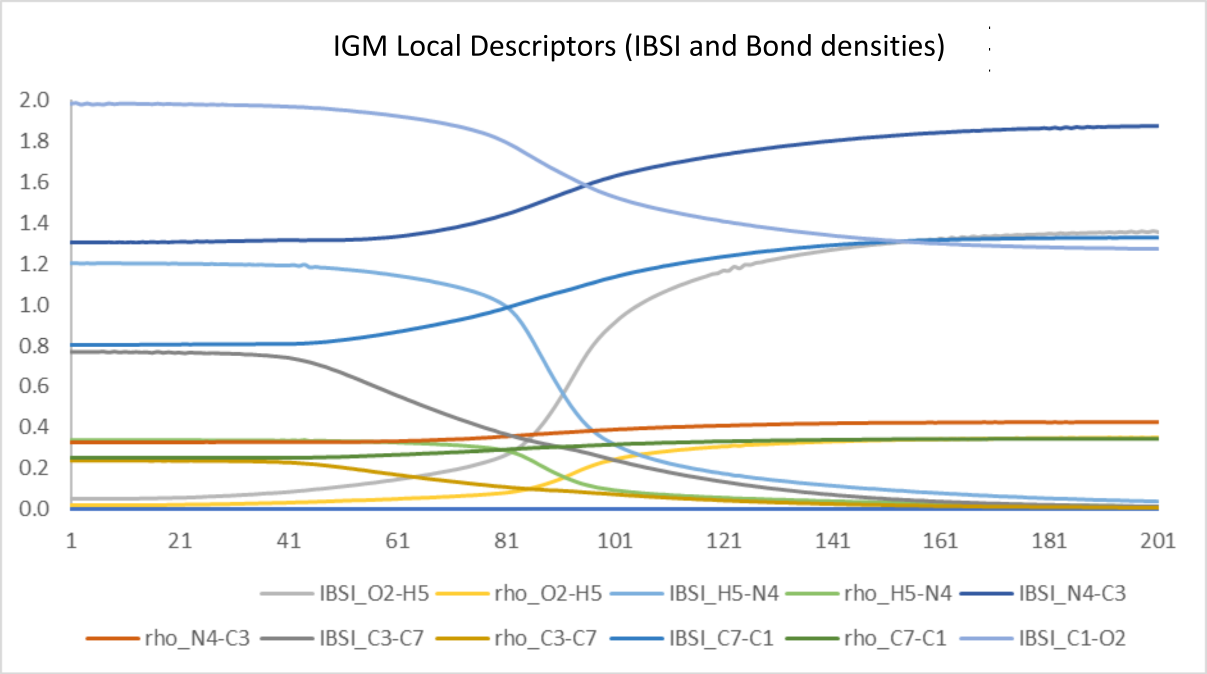


**Figure S2.** Calculations of local descriptors using electron density (rho) and IGM theory of compound 12, GD3BJ/Def2TZVP at 450 K. 201 points were used and at each point both the electron density of the bond and its strength were determined through the IBSI index.

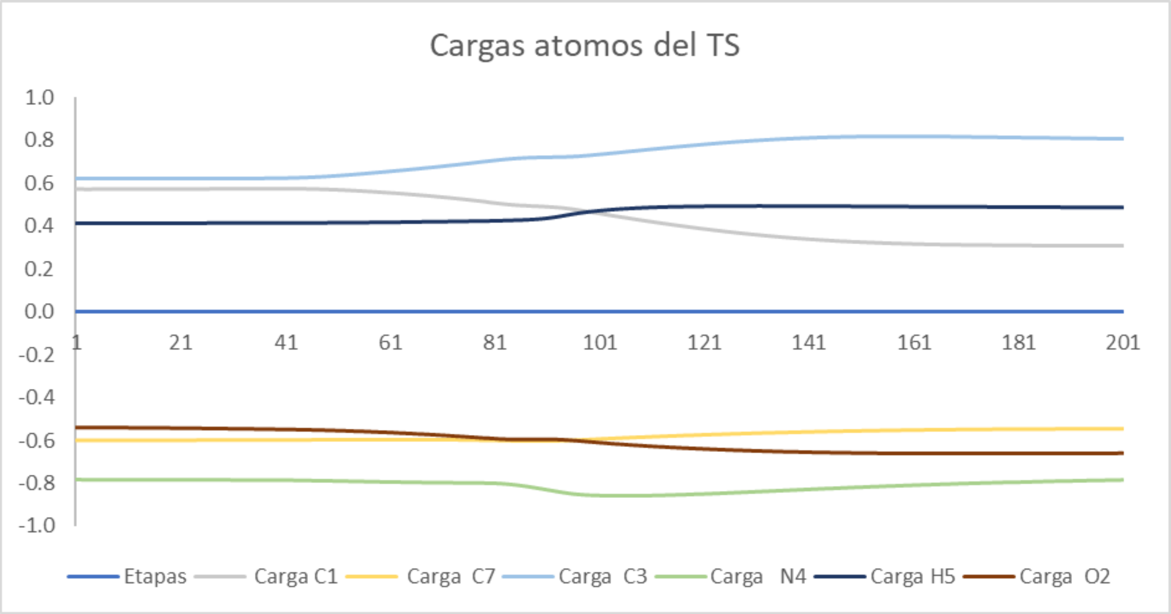


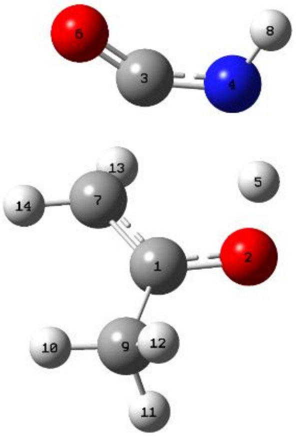


**Figure S3.** Calculations of the NBO charges of the atoms involved in the transition state, as shown above. Compound 12, GD3BJ/Def2TZVP at 450 K, was used as a base.

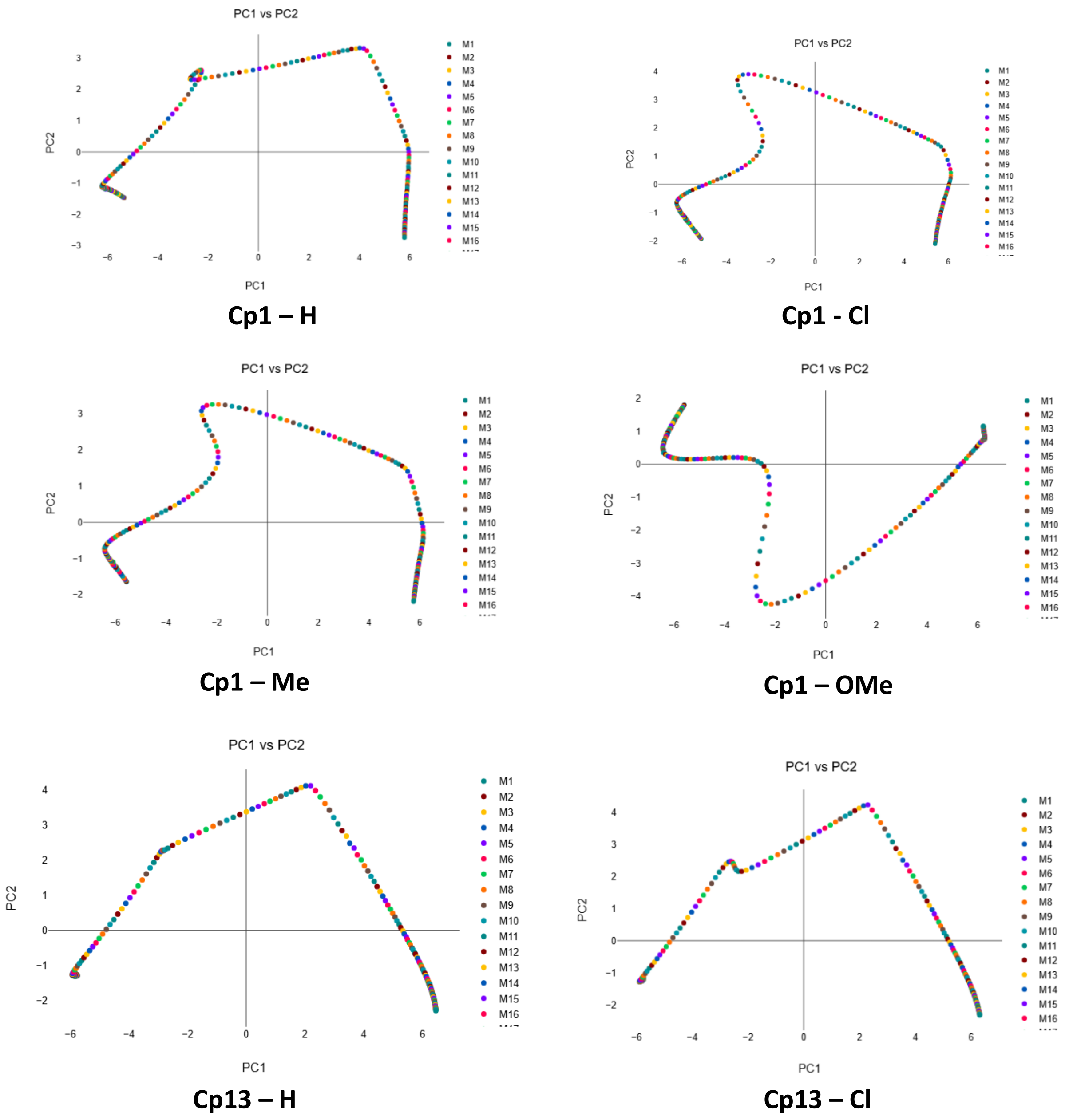


Figure S4. Graph of scores of the principal components (PC1 vs PC2) of the studied compounds, resulting from the 201 IRC points, showing the separation between more reactive (PC1 > 0) and less reactive (PC1 < 0) structures along the reaction coordinate.

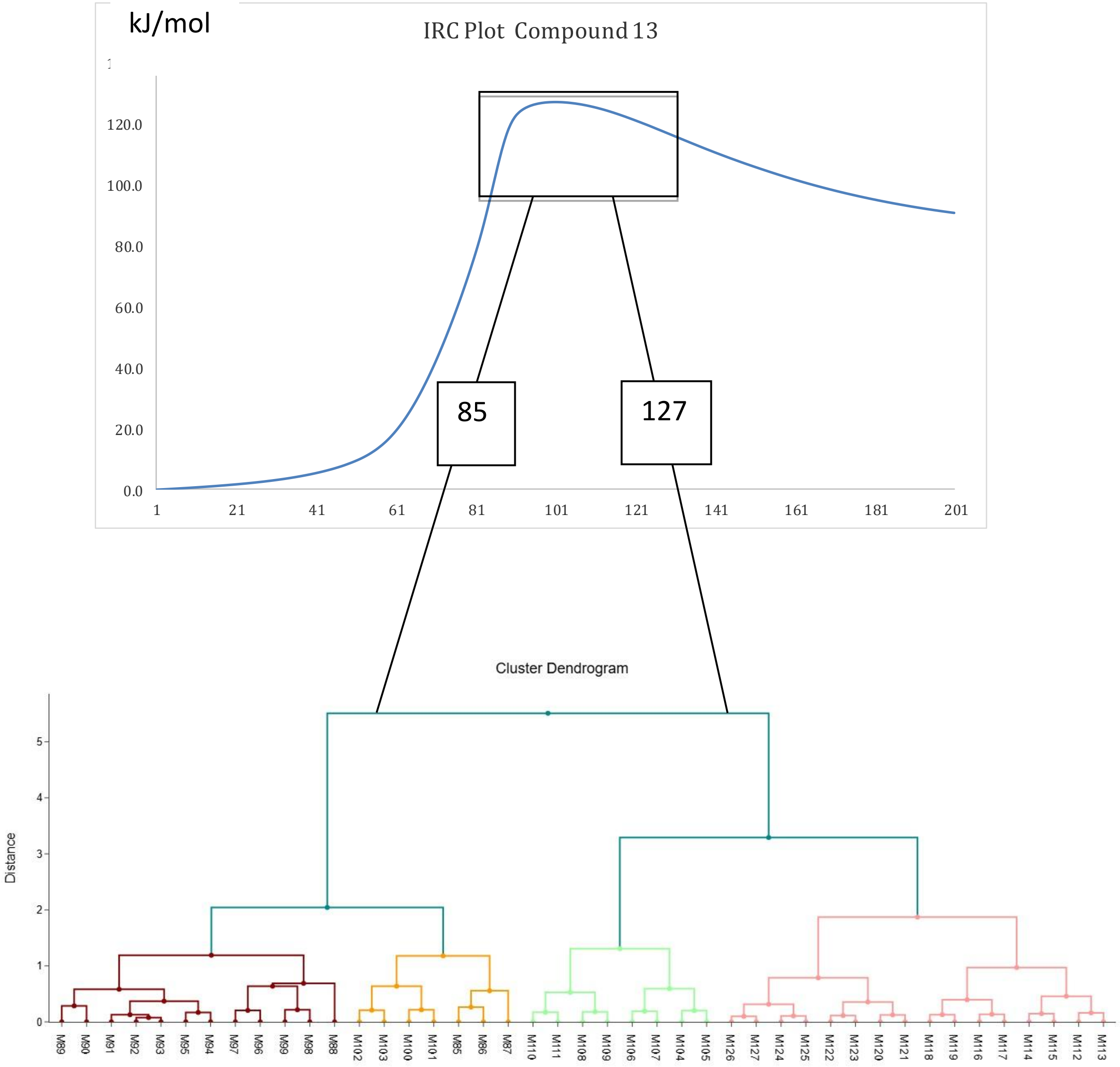


Figure S5. Dendrogram of the hierarchical cluster analysis (HCA) for Compound 13 (N-phenyl-3-oxo-3-phenylpropanamide), based on 61 CRI points (M85–M127) and using the 30 DFT variables (global descriptors, IBSI, electron densities and NPA loads) at the B97D-GD3BJ/ Def2TZVP level at 450 K. The transition state (TS) is located at M101. The dendrogram shows a distribution of the dots that reflects the effect of the additional phenyl group on the dynamics of the reaction, with a reorganization of the clusters with respect to the reference compound (Figure 8).
